# An Updated Comprehensive Review of Plants and Herbal Compounds with Antiasthmatic Effect

**DOI:** 10.1155/2024/5373117

**Published:** 2024-02-08

**Authors:** Mohammad Amin Rajizadeh, Hamid Najafipour, Mohammad Abbas Bejeshk

**Affiliations:** ^1^Physiology Research Center, Institute of Neuropharmacology, Kerman University of Medical Sciences, Kerman, Iran; ^2^Cardiovascular Research Center, Institute of Basic and Clinical Physiology Sciences, Kerman University of Medical Science, Kerman, Iran; ^3^Endocrinology and Metabolism Research Center, Institute of Basic and Clinical Physiology Sciences, Kerman University of Medical Sciences, Kerman, Iran

## Abstract

**Background:**

Asthma is a common disease with rising prevalence worldwide, especially in industrialized countries. Current asthma therapy with traditional medicines lacks satisfactory success, hence the patients' search for alternative and complementary treatments for their diseases. Researchers have conducted many studies on plants with antiallergic and antiasthmatic effects in recent decades. Many of these plants are now used in clinics, and searching for their mechanism of action may result in creating new ideas for producing more effective drugs.

**Purpose:**

The goal of this review was to provide a compilation of the findings on plants and their active agents with experimentally confirmed antiasthmatic effects. *Study Design and Method*. A literature search was conducted from 1986 to November 2023 in Scopus, Springer Link, EMBASE, Science Direct, PubMed, Google Scholar, and Web of Science to identify and report the accumulated knowledge on herbs and their compounds that may be effective in asthma treatment.

**Results:**

The results revealed that 58 plants and 32 herbal extracted compounds had antiasthmatic activity. Also, 32 plants were shown to have anti-inflammatory and antioxidative effects or may act as bronchodilators and potentially have antiasthmatic effects, which must be investigated in future studies.

**Conclusion:**

The ability of herbal medicine to improve asthma symptoms has been confirmed by clinical and preclinical studies, and such compounds may be used as a source for developing new antiasthmatic drugs. Moreover, this review suggests that many bioactive compounds have therapeutic potential against asthma.

## 1. Introduction

Asthma is a chronic inflammatory change in the pulmonary system [1]. The airway hyperresponsiveness (AHR) that results from this chronic inflammation leads to recurrent episodes of asthma symptoms, which can vary in intensity over time [1]. Asthma is an obstructive pulmonary disease with different etiologies, affecting approximately 300 million people worldwide [2, 3]. By 2025, a further 100 million will be added to this estimate [4]. In the USA alone, the annual cost of asthma is approximately $56 billion. The significant impact of airway inflammation in the pathophysiology of asthma has been confirmed by many clinical and basic studies [5]. The chronic airway remodeling, especially thickening of airway walls, and inflammation is mediated by potent chemical mediators [6]. The disease progression and chronicity is further aggravated by the resulting narrowing of airways [7, 8]. The primary goal of asthma management is the reduction of disease prevalence and mortality through maintaining control over the disease in order to prevent exacerbations. Therapy also aims to improve lung function and overall quality of life, normalize physical activity, reduce the necessity of medicine, and improve the symptoms in terms of quantity and quality [9]. Medications that are currently used to relieve asthma symptoms in the clinic are inhaled corticosteroids (ICSs), leukotriene receptor antagonists (LTRAs), long-acting beta-agonists (LABAs) in combination with an ICS, long-acting muscarinic receptor antagonists (LAMAs), monoclonal antibody immune-modulating drugs, mast cell stabilizing medications, methylxanthine drugs, and biologic substances, such as anti-IL-5 therapy and anti-IgE therapy. Medications used for asthma relief include inhaled anticholinergic drugs and rapid-acting beta2-agonists [10]. The drugs that are currently prescribed have several side effects that can vary depending on the drug class, its dose, and how it is administered. Some side effects of beta-agonists include cough, headache, vomiting, fever, nausea, sweating, dyspepsia, and increased insomnia and nervousness in children [11]. The systemic anticholinergic activity of LAMAs causes side effects such as lower urinary tract symptoms and urinary retention, especially in older men, dizziness, and excessive dry mouth [12]. Common side effects that may occur with ICSs include headache, throat irritation, dysphonia, cough, viral gastroenteritis, and back and oropharyngeal pain [13]. LTRAs cause other issues, including abdominal pain, headache, dizziness, dyspepsia, elevated liver function indicators, and cholestatic hepatitis [11, 13]. Plants and natural sources contribute largely to the commercial drug preparations manufactured today [14–16]. Traditional and plant-derived compounds are the treatment of choice for most (>80%) of the global population, and about 25% of prescriptions in the United States contain at least one plant-derived ingredient [17–19]. Therefore, consumers should be given science-based information on dosage, contraindications, and efficacy [20–22]. If sufficient scientific evidence of the benefits of an herb is available, the benefits can be employed to promote public health and treat disease [23–25]. There are recent reviews about herbal plants used for asthma control. However, these reviews generally focus on a specific asthma model or class of herbs, such as traditional Chinese or Indian herbs [26]. Since there is no definite treatment for asthma and the drugs available in the clinic mostly relieve the symptoms of the disease and the drug's side effects, delineating the role of herbal compounds in improving and treating asthma can be helpful. In this comprehensive review, we searched the literature as far back as 37 years ago for information about herbal plants and herbal compounds that have shown real or potential antiasthmatic activity. Also, the studies that have pointed out these properties for each plant or compound are introduced along with the probable mechanisms of action to help the researchers continue the earlier studies towards producing new and effective drugs with fewer adverse effects against asthma.

## 2. Method

We used the searched terms “asthma,” “the plants affecting the respiratory system,” “phytomedicine,” “herbal compounds,” “alkaloids,” “polyphenols,” “flavonoids,” “medicinal plants/herbs,” “plants,” and “herbs,” in the Scopus, Springer Link, EMBASE, Science Direct, PubMed, Google Scholar, and Web of Science databases. All the studies related to the above keywords were assessed. Articles published from 1986 to November 2023 were included. In this review, preclinical (animal studies, *in vitro* experiments) and clinical investigations (clinical trials) were considered. The main criteria for selecting herbs and compounds were their anti-inflammatory, antiasthmatic, antioxidative, antiremodeling, antihistaminic, and anticholinergic effects. Inclusion and exclusion criteria were established to select articles that met the specific research question. The titles and abstracts of the identified articles were reviewed to identify those relevant to the research question. The full texts of the selected articles were then carefully assessed to determine their inclusion in the review. The search terms were clustered according to the PICO (Population, Phenomenon of Interest, Context) scheme to ensure a systematic approach. This allowed for the inclusion of research examining the impact of herbs or their active agents on asthma control and treatment. Nevertheless, because of large number of papers found, to reduce the length of the review, in cases where more than one paper reported an individual property of a plant (for example, the anti-inflammatory effect), only the more recent/more comprehensive study was cited.

## 3. Results

**Figure 1:**
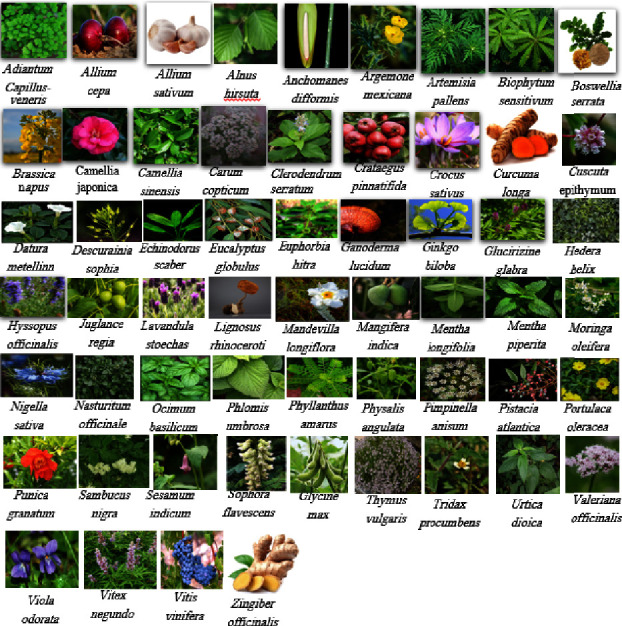
Pictures of plants with verified antiasthmatic effects. The plant names are arranged in alphabetical order. The same ordering method is used in the text when describing the plant characteristics.

The results revealed that over 120 plants or their extracted compounds have shown antiasthmatic, antiremodeling, antiapoptotic, anti-inflammatory, antioxidative, antihistaminic, anticholinergic, and bronchodilator activity. The description of the herbs (58 herbs) or their compounds (32 compounds) found with antiasthmatic activity are presented in alphabetic order in the text. The findings are summarized in two tables at the end of the paper. The effects on pulmonary function tests or the airway responsiveness corresponding to clinical studies are shown in Table 1, and the effects on inflammatory, oxidative, remodeling, cholinergic, bronchodilatory, and histaminic characteristics corresponding to preclinical studies are shown in Table 2. The pictures of the plants with verified antiasthmatic activity are presented in Figure 1 (58 plants), and the pictures of those with potential antiasthmatic activity are presented in Figure 1 of the supplementary file (available here) (32 plants).

### 3.1. Plants with Verified Antiasthmatic Activity

Plants that have shown antiasthmatic activity are described first in alphabetic order. In many of these studies, the effects of plants have been compared with medicines currently used in the clinic. These plants are of different species, and the mechanism of action of most of them against asthma is to inhibit inflammation and oxidative stress.

#### 3.1.1. *Adiantum capillus-veneris* L. (Fern)

The venus hair fern, maidenhair fern, black maidenhair fern, and southern maidenhair fern are species of ferns in genus *Adiantum* and family *Pteridaceae* distributed in subcosmopolitan areas worldwide. In general, the ameliorative effects of this plant have been shown in respiratory disorders, including asthma [147]. It has been shown that its leaf ethanolic extract in concentrations of 250 and 500 mg/kg has antiasthmatic (H1 receptor antagonist) activity in experimental models of asthma [51].

#### 3.1.2. *Allium cepa* (Onion)

The onion (*Allium cepa* L., from Latin *cepa*, “*onion*”) is a vegetable that is the most widely cultivated species of the *Allium* genus [148]. Marefati et al. showed that *Allium cepa* extract, at concentrations of 35, 70, and 140 mg/kg, significantly decreases IL-4, IgE, and oxidant markers and elevates antioxidant markers (SOD, CAT, and thiol), IFN-*γ*, and IFN-*γ*/IL-4 ratio in comparison to 1.25 *μ*g/mL dexamethasone in asthmatic rats [52]. Moreover, another study revealed that *Allium cepa* extract has a potential therapeutic effect on asthmatic mice by reducing bronchoalveolar lavage fluid (BALF) IL-13, IL-5, and IL-4 [53]. Shakeri et al. showed the antiasthmatic effects of this plant at the concentration of 0.7 mg/ml by inhibiting MDA, NO_2_, NO_3_, and total protein in rats [149]. The extract has been shown to reduce the number of bronchus mast cells of an asthmatic model caused by ovalbumin at 140 mg/kg [150].

#### 3.1.3. *Allium sativum* (Garlic)

Garlic *(Allium sativum)* is a species in the onion genus *Allium*. It is native to Central Asia and northeastern Iran. El-Din et al. showed that *Allium sativum* is effective against the severe pathological defects of lambda cyhalothrin-induced asthma in rats. *Allium sativum* at the concentration of 100 mg/kg has been found to alleviate pesticide-induced obstruction of bronchi (noneosinophilic asthma) with interstitial alveolitis and formation of abscess in albino rats [151]. It has been shown that garlic's sulfur compounds benefit asthma patients in various ways, through the modulation of antiviral cytokines and inflammation and other antiviral, antibacterial, and antioxidant mechanisms. As the development of asthma is delayed this way, this treatment may be considered an alternative or coadjutants in the treatment of asthma [152].

#### 3.1.4. *Alnus hirsuta* (Spach)

In Eastern Asia, alcoholism, diarrhea, and hemorrhage are treated with compounds derived from this member of the *Betulaceae* family. Using an ovalbumin-induced murine asthma model to compare *Alnus hirsuta* with dexamethasone (3 mg/kg), Lee et al. found that this plant alleviates mucus overproduction and airway inflammation at the concentration of 50 mg/kg [153].

#### 3.1.5. *Anchomanes difformis* (Blume)

In Delta State, Nigeria, herbal practitioners reportedly use *Anchomanes difformis* to treat asthma [154]. Its leaf aqueous extract was compared with salbutamol as reference in guinea pigs, and the difference in antiasthmatic activity between the aqueous leaf extract (400 mg/kg) (32.7%) and salbutamol (32.5%) was not significant. The experiment verified the asthma-relieving effect and safety of *A. difformis* leaf extract [30, 155].

#### 3.1.6. *Argemone mexicana*

This plant grows abundantly in India [156]. *A. mexicana* stem aqueous extract, at a concentration of 50 mg/kg i.p., possesses antiallergic and antistress activity in milk-induced leukocytosis and eosinophilia [156, 157]. This plant's oxidation- and inflammation-relieving effects are well known [158]. Singh et al. stated that the ethanolic extract of *Argemone mexicana* stems at concentrations of 150, 250, and 350 mg/kg has antiasthmatic activity via reducing eosinophil count and protection against bronchospasm [27].

#### 3.1.7. *Artemisia pallens*

*Artemisia pallens* is an aromatic herb in the genus of small herbs or shrubs. Mukherjee et al. demonstrated the antiasthmatic potential of 200 and 400 mg/kg *Artemisia pallens* in OVA-induced alveolar hyperresponsiveness (AHR) in rats compared to montelukast (10 mg/kg) via a plethora of mechanisms [54]. These include inhibition of oxidonitrosative stress (reducing MDA, MPO, and NO and increasing SOD and GSH) and reduction of IgE, TGF-*β*, TNF-*α*, IL-4, IL-1*β*, and IL-6. It also increases Nrf2 levels and improves pulmonary function tests in rats [54].

#### 3.1.8. *Biophytum sensitivum*

*Biophytum sensitivum* 100 and 200 mg/kg leaf alcoholic extract (p.o.) has been investigated for its antiasthmatic effects in guinea pigs. The 200 mg/kg extract showed the maximum percentage of protection (67.5%) compared to the standard drug chlorpheniramine maleate (1 mg/kg, p.o.) (76.6% protection) in the same time interval. The investigation confirmed that by inhibiting the bronchoconstriction induced by histamine, the extract exerts significant antiasthmatic effect [30].

#### 3.1.9. *Boswellia serrata* (Frankincense)

Indian frankincense is produced using this plant. A clinical study in 2010 demonstrated that the extract has a pronounced effect on managing bronchial asthma, significantly decreasing plasma NO, MDA, and LTC4 levels [62]. In a randomized open-label comparative clinical study, *Boswellia* significantly decreased asthmatic attack episodes and increased forced vital capacity (FVC) values and forced expiratory volume in the first second (FEV1) [31, 159]. There are old placebo-controlled double-blind clinical studies where bronchial asthma patients were treated for six weeks, three times daily, with either lactose as placebo or gum resin (300 mg). Decrease in erythrocyte sedimentation rate (ESR) and eosinophilic count, elevation in peak expiratory flow rate, FVC, and FEV1, and alleviation of disease physical symptoms and signs (frequency of attacks, rhonchi, and dyspnea) were recorded in 70% of the treatment group but only in 27% of the placebo group [32, 159].

#### 3.1.10. *Brassica napus* (Rapeseed)

Rapeseed *(Brassica napus* subsp. *napus)* is a bright yellow flowering member of the family *Brassicaceae* (mustard or cabbage family), which naturally contains appreciable amounts of erucic acid. Neamati et al. disclosed that *Brassica napus* L. oil at 0.5 and 0.75 mg/kg could decrease lung eosinophil count and airway smooth muscle thickness in asthmatic rats [63].

#### 3.1.11. *Camellia japonica*

*Camellia japonica* is from the *Theaceae* family. Recently, its biological functions, such as its oxidation- and inflammation-reducing effects, have been reported. Lee et al. demonstrated that *Camellia japonica* oil at 100 and 500 mg/kg doses suppresses asthma occurrence via the GATA3 and IL-4 pathways compared to dexamethasone (10 mg/kg). They reported that it reduces IgE, eosinophil count, and WBC in BALF. Also, it diminishes mucous hypersecretion, epithelial cell hyperplasia, inflammatory cell infiltration, and IL-4, IL-5, IL-6, IL-13, and TNF-*α* in the lungs of asthmatic mice [64].

#### 3.1.12. *Camellia sinensis* (Tea)

The leaves and leaf buds of these small evergreen trees or shrubs of the *Theaceae* family are used to produce tea. Heo et al. demonstrated that the aqueous extract of *Camellia sinensis* at the concentration of 25 *μ*g/ml exerts antiasthmatic activity by alleviating asthma-related cytokine activity through increasing the expression of TGF-*β*, IFN-*γ*, and IL-10 and by decreasing the expression of inflammatory cytokines IL-4, IL-13, and IgE in the lungs [65]. Some studies have revealed that mucus hypersecretion and airway inflammation are regulated by tea extract, improving allergic asthma induced by ovalbumin [160, 161].

#### 3.1.13. *Carum copticum* (Ajwain)

The seed of Ajwain is a member of the *Apiaceae* family, believed to have a hot humor, and is commonly used in Indian cuisine [33]. The strong bronchodilatory effect of 0.125 and 0.25 ml/kg *C. copticum* on asthmatic airways was demonstrated in a clinical study by Boskabady et al., an effect comparable with that of theophylline (6 mg/kg) [162]. They reported a significant increase in lung function tests (sGaw, MEF25, MEF50, MEF75, MMEF, PEF, and FEV1) between 30 and 150 min post administration of high concentrations of its boiled extract [162].

#### 3.1.14. *Clerodendrum serratum* Linn. (*Verbenaceae*)

Fever, respiratory diseases, rheumatism, inflammation, and pain are traditionally treated with *Clerodendrum serratum* (bharangi in Ayurveda alternative medicine). *C. serratum* root alcoholic extract has been demonstrated to possess antiasthmatic effects in isolated goat tracheal chain preparation and antieosinophilia in mice at concentrations of 50, 100, and 200 mg/kg [163]. This plant's antiasthmatic, anti-inflammatory, antioxidant, antihistaminic, and antiallergic features are well recognized [164, 165]. The protective effects of 100 mg/kg icosahydropicenic acid isolated from the roots of *C. serratum* (L.) Moon on experimental allergic asthma was reported by Bhujbal et al. [166]. The reference drug was sodium cromoglycate (50 mg/kg). Kumar et al. revealed that aqueous and nonaqueous extracts of *C. serratum* have antiasthmatic effects in rats [167]. Arora et al. found that the extract reduces the inflammation-inducing mediators in ovalbumin-induced asthma in rats [168].

#### 3.1.15. *Crataegus pinnatifida* (Mountain Hawthorn)

Administration of *Crataegus pinnatifida* ethanolic extract at concentrations of 100 and 200 mg/kg in the murine asthma model significantly reduces inflammatory cells, especially eosinophils in the lung tissue and BALF and decreases AHR, OVA-specific IgG levels, OVA-specific IgE, and total IgE in the serum and also eotaxin, IL-13, IL-5, and IL-4 after OVA challenge in BALF in comparison with montelukast (30 mg/kg). These findings indicate that *Crataegus pinnatifida* ethanolic extract may be effective in inhibiting the development of airway inflammation in allergic asthma. In addition, the alcoholic extract of *Crataegus pinnatifida* exerts its anti-inflammatory effect partially through downregulating MMP-9, leading to a decrease in the expression of VCAM-1 and ICAM-1 [37].

#### 3.1.16. *Crocus sativus* (Saffron)

Pharmacological investigations have confirmed that the extract or the constituents of this stemless perennial member of the *Iridaceae* family [169] have free radical-scavenging effects and antioxidant properties [170], and relaxant effects on tracheal smooth muscles [70, 170]. Previous studies have shown that this plant inhibits histamine (H1) receptors [71] and stimulates *β*-adrenoceptors [171]. Mahmoudabady et al. showed the ability of *C. sativus* extract to reduce eosinophils and other inflammatory cells in the lung lavage of sensitized animals [172]. Safranal, the main constituent of *C. sativus*, attenuates AHR and airway structural changes during allergic asthma and decreases IL-13 and IL-5 and other inflammatory cytokines in mouse lungs [38]. It has been shown that safranal and saffron alleviate OVA-induced asthma, inhibit mast cell activation, and reduce serum endothelin [173]. Oral administration (4, 8, and 16 mg/mL) of *C. sativus* extract to OVA-sensitized guinea pigs resulted in a reduction in total and differential WBC in blood, a rise in serum IFN-*γ*/IL-4 ratio and IFN-*γ*, and a reduction in TP and ET-1 serum levels. However, serum levels of IL-4, total NO, nitrite, and tracheal responsiveness decreased. The reference drug was 50 mg/mL dexamethasone [174]. A double-blind, randomized, placebo-controlled clinical study demonstrated the beneficial effects of 100 mg of saffron daily on asthma symptom severity [175].

#### 3.1.17. *Curcuma longa* Rhizome (Turmeric)

Curcumin is a bright yellow chemical produced by *Curcuma longa* plants (turmeric), a member of the ginger family Zingiberaceae. The results of Moon et al. in 2008 demonstrated that 10 or 20 mg/kg curcumin can suppress AHR and infiltration of inflammatory cells in the lung, decrease iNOS expression, attenuate the expression of IL-4 and IL-5 in the BALF and IgE in the serum, and inhibit NO expression in lung epithelial cells of asthmatic BALB/C mice induced by OVA [73]. Another study showed that curcumin, at concentrations of 50, 100, and 200 mg/kg, in comparison to dexamethasone (2 mg/kg), effectively suppressed IL-17A, improved IL-10, and inhibited both the recruitment of eosinophils and mucus overproduction in mice with OVA-induced asthma. Curcumin attenuated allergic airway inflammation by regulating CD4+ CD25+ Tregs/Th17 balance in OVA-sensitized mice [176]. Gao et al. revealed that after eight weeks of treatment with curcumin, at a concentration of 200 mg/kg, brought about significant improvement in the areas of the aortic root that had lesions, and the Th17 and Th2 cells that had been elevated showed significant decrease [177]. According to another study, postbronchodilator FEV1 in atopic asthma patients was not significantly affected by oral curcumin administration [40]. It has been shown that curcumin administered intranasally modulates MAPK signaling and NF-*κ*B activation, regulating chronic asthma in mice as a result [178, 179]. In contrast, Abidi et al. revealed that curcumin is effective and safe as an add-on therapy for the treatment of bronchial asthma [180]. Curcumin administered intranasally prevented remodeling related to chronic asthma, structural alterations, airway inflammatory cell accumulation, and mucus secretion in the OVA-induced murine model of chronic asthma, along with a decrease in IL-5, IL-4, and IgE levels [74, 181]. Yang et al. demonstrated that a mouse asthma model, lung inflammation is reduced by curcumin through Wnt/*β*-catenin signaling [182]. Curcumin incorporated into a liposome can suppress the TNF-*α*, IL-1*β*, IL-6, and IL-8 proinflammatory markers in asthmatic animals [75]. Boskabady et al. showed the ameliorative effect of curcumin at concentrations of 0.15, 0.30, and 0.60 mg/ml on serum levels of CAT, MDA, NO_3_, NO_2_, and SOD, total and differential WBC, lung pathological changes, and tracheal responsiveness in the animal asthmatic model, comparable or more potent than dexamethasone [183]. Liu et al. suggested that curcumin effectively alleviates airway inflammation in asthmatic mice and downregulates the expression of proinflammatory cytokines, probably through the Nrf2/HO-1 signaling pathway and reduction in IL-6, IL-1*β*, and TNF-*α* [76]. Zhu et al. revealed that through a PPAR*γ*-dependent NF-*κ*B signaling pathway, curcumin improves mucus hypersecretion and asthmatic airway inflammation *in vitro* and *in vivo* [184].

#### 3.1.18. *Cuscuta epithymum* (*Cuscuta chinensis* Lam.)

This plant is a yellow, rootless, apparently leafless perennial grapevine often found in tropical and temperate regions, includingIndia and China. Some species are widely used to treat asthma. *C. chinensis* seed methanol extract can reduce IL-6, TNF-*α*, NF-*κ*B, COX-2, and IL-1*β* in asthma [185].

#### 3.1.19. *Datura metel* Linn

Known in the U.S.A. as angel's trumpet or devil's trumpet and in Europe as metel, Hindu datura, or Indian thornapple, *Datura metel* Linn. has been shown to ameliorate asthma symptoms at a concentration of 0.56 mg/kg in Balb/c mice by reducing the number of activated T cells and maintaining T cells in naïve-type status [186].

#### 3.1.20. *Descurainia sophia*

*Descurainia sophia* is a member of the family *Brassicaceae.* Pi et al. showed that through the regulation of epithelial damage and airway inflammation, *Descurainia sophia* improves lung permeability in rats with allergic asthma [187].

#### 3.1.21. *Echinodorus scaber* Rataj

This is the heterotypic synonym for *Echinodorus macrophyllus* (Kunth) of the *Alismataceae* family. Rosa et al. showed that the hydroethanolic leaf extract from this subaquatic herbaceous Brazillian native reduces inflammation in OVA-induced allergic asthma through decreasing BALF IL-13, IL-5, IL-4, and IgE at concentrations of 1, 5, and 30 mg/kg, which is comparable with dexamethasone (0.5 mg/kg) [188].

#### 3.1.22. *Eucalyptus globulus*

Known as rose or flooded gum, *Eucalyptus grandis* is a tall smooth tree, which is rough at the base. Soyingbe has shown the anticough and antiasthmatic qualities of *Eucalyptus grandis* [189]. In a clinical trial, Juergens et al. reported that 1.8-cineol (eucalyptol) exerts anti-inflammatory effects at 600 mg/day in bronchial asthma by demonstrating improvement in lung function tests [190].

#### 3.1.23. *Euphorbia hirta*

A member of the family *Euphorbiaceae*, *Euphorbia hirta* is a well-known name in traditional medicine. The anti-inflammatory properties of this plant are well demonstrated [191]. Xia et al. revealed that *E. hirta* extract administration at concentrations of 1 and 2 *µ*g/*µ*l reduces total leukocytes, eosinophils, lipid peroxidation, IL-6, and TNF-*α* but increases antioxidant levels in asthmatic rats. Also, the mRNA expression levels of TNF-*α*, iNOS, Bax, nerve growth factor precursor, p53, caspase-3, cyclooxygenase-2 (COX-2), IL-6, and caspase-3 decrease, whereas Bcl2 expression level increases in asthmatic rats following treatment [192].

#### 3.1.24. *Ganoderma lucidum*

Liu et al. showed that ganoderic acid C1 (GAC1) isolated from *G. lucidum* inhibits TNF-*α* production in asthma patients' peripheral blood mononuclear cells (PBMCs) and in murine macrophages stimulated by LPS and associated this effect with NF-*κ*B signaling pathway suppression and, in part, with MAPK and AP1 pathway suppression. Inflammatory diseases such as TNF-*α*-associated asthma may be treatable by GAC1 as a novel therapy [193].

#### 3.1.25. *Ginkgo biloba*

*Ginkgo biloba*, commonly known as ginkgo or gingko, is the only living species in the division *Ginkgophyta*, all others having gone extinct [88]. According to the results of animal studies, administration of *G. biloba* at concentrations of 100 and 150 mg/kg diminishes the number of goblet cells and mast cells, epithelium thickness, subepithelial smooth muscle thickness, and basement membrane thickness in asthmatic mice [89], and reduces IL-4 and IgE levels at 12.5 mg/kg in asthmatic rabbits (compared with 0.5 mg/kg prednisolone) [90]. It also decreases leukocytes, eosinophils, IL-4, IL-13, and TNF-*α* levels in the BALF of asthmatic mice [91]. Chu et al. found that at 40 mg/kg dose, *ginkgolide B* inhibited the ERK/MAPK signaling pathway, exerting anti-inflammatory effects in asthma [194]. A clinical study showed that treatment with *Ginkgo biloba* extract results in reduced blood platelet activating factor in asthmatic children and improves asthma symptoms [195]. Zheng et al. showed that *Ginkgo biloba* tablets added to routine therapy can improve lung function (FEV1/FVC) in steroid-dependent asthma patients [196]. Yijune et al. in a clinical study reported that the *Ginkgo biloba* extract could significantly decrease the infiltration of inflammatory cells in the asthmatic airways, and it may be used as a complement to glucocorticosteroid therapy [197].

#### 3.1.26. *Glycirizine glabra*

Liquorice is the common name of *Glycyrrhiza glabra*, a flowering plant of the bean family, *Fabaceae* [92]. Hocauglu et al. demonstrated that glycyrrhizin diminishes goblet cells and mast cells, epithelium thickness, subepithelial smooth muscle thickness, and basement membrane thickness at 10 mg/kg in the mouse asthma model, benefiting all long-term histopathologic changes of the lung, comparable with dexamethasone (1 mg/kg) [44]. A clinical study showed that oral administration of 50 mg soft-gelatin capsules of licorice extract (6.5% *glycyrrhizin*) three times a day for four weeks effectively reduces frequency and severity of asthma attacks [62]. Ram et al. showed that *Glycyrrhiza glabra* at concentrations of 2.5, 5, 10, and 20 mg/kg significantly alleviates the asthmatic symptoms by an increase in IFN-*γ* and reduction in BALF eosinophil count, IL-5, and IL-4 in comparison with dexamethasone (1 mg/kg) [93]. Khattab et al. found that licorice extract at the concentration of 40 mg/kg prevents the production of Th2 cytokines and free radicals induced by ovalbumin in mice via raising CAT and SOD levels and reducing IgE, IL-5, IL-13, NO, and MDA levels in plasma and BALF in comparison to montelukast (30 mg/kg) [43]. Yang et al. demonstrated the synergistic antiasthmatic effects of glycyrrhizinand salbutamol (*β*2-agonist) in the treatment of asthma [198]. Chen et al. showed the effect of glycyrrhizic acid loaded on PLGA nanoparticles in treating allergic asthma [199]. A clinical study showed that aqueous liquorice extract capsules significantly improve pulmonary function tests (increase in FVC% and FEV1%) in asthmatic patients [200].

#### 3.1.27. *Hedera helix* (Ivy)

*Hedera helix*, the common ivy, is a member of the *Araliaceae* family [201]. Zeil et al. have demonstrated that the lung function parameters of children with uncontrolled, persistent, mild asthma experience significant improvement after combined therapy of ivy leaf extract and inhaled corticosteroids (budesonide) [201]. Babayigit Hocaoglu et al. showed that *Hedera helix* administration at the concentration of 100 mg/kg reduces goblet cell numbers and thickness of basement membrane in the lung of asthmatic mice, comparable to 1 mg/kg dexamethasone [202]. A systematic review in 2011 by Holzinger et al. approved the effectiveness of ivy leaves for treating acute upper respiratory tract infections [203]. Another clinical study revealed that ivy leaf extract improves the respiratory functions of children with chronic asthma [204].

#### 3.1.28. *Hyssopus officinalis* (Zufa)

The medicinal plant *Hyssopus officinalis* L., which belongs to the family *Lamiaceae*, is widely cultivated in Asia, Europe, and temperate regions of America [97]. Ma et al. showed that *Hyssopus officinalis* at concentrations of 0.04 g/kg and 1.6 g/kg regulates the MMP-9/TIMP-1 ratio via affecting the expressions of some cytokines (such as IL-1, IL-17, and TNF-α) and improves airway remodeling in asthmatic mice compared to dexamethasone (5 mg/kg) [98]. Another study showed that *Hyssopus officinalis* could inhibit the secretion of IL-17 in serum and solve Th1/Th2 cytokine (IL-4 and IFN-*γ*) imbalance in asthmatic rats [205]. A clinical study showed that *Hyssopus officinalis* effectively relieves the signs and symptoms of bronchial asthma [100]. Fengjuan et al. showed that JAX2, an ethanol extract of hyssop, could reduce BALF IgE, TNF-*α*, IL-17, IL-6, and IL-4 and inhibit MAPK/NF-*κ*B inflammatory signaling, resulting in the prevention of bronchial asthma in rats [206].

#### 3.1.29. *Juglans regia*

Also known as white or English walnut, *Juglans regia* is used by Palestinians to treat asthma and diabetes. Sharif et al. showed that by upregulating aquaporin-5 and aquaporin-1 and downregulating inflammatory cytokines, *Juglans regia* ameliorates allergic asthma caused by ovalbumin exposure in mice [207].

#### 3.1.30. *Lavandula stoechas*

Known as French lavender (U.K.) or topped or Spanish lavender (U.S.), *Lavandula stoechas* is a member of the *Lamiaceae* family. Almohawes et al. confirmed that *Lavandula* at the concentration of 300 mg/kg has antiasthma, anti-inflammatory, and antioxidant activity through reducing serum IgE and IgG concentration, diminishing MDA, and increasing SOD in asthmatic guinea pigs [104, 208]. Khodadoost et al. revealed the alleviating effects of *Lavandula* aqueous extract at the concentration of 833 mg/kg on asthma complications via reducing eosinophils and IL-5, IL-33, and IL-13 concentration in BALF in a mouse model in comparison to budesonide (5 mg/kg) [209].

#### 3.1.31. *Lignosus rhinocerotis*

*Lignosus rhinocerotis* Cooke is a medicinal mushroom traditionally used by indigenous communities in Malaysia to treat asthma and several other diseases. It has been shown that *Lignosus rhinocerotis* Cooke reduces airway hyperresponsiveness, hypersecretion of mucus, and inflammation of airways at the concentration of 125 mg/kg in a murine asthmatic model compared to the dexamethasone (3 mg/kg) [210].

#### 3.1.32. *Mandevilla longiflora*

*Mandevilla longiflora*, popularly known as “velame” in central Brazil, is a subshrub widely distributed in South America. Almeida et al. found that at concentrations of 20, 50, and 200 mg/kg, *Mandevilla longiflora* reduces inflammatory cells, IL-13, IL-5, IL-4, IgE, and LT-B4 in BALF, improving airway inflammation in the murine model of allergic asthma comparable to dexamethasone [105].

#### 3.1.33. *Mangifera indica*

Also known as the mango, this member of the poison ivy and sumac family (*Anacardiaceae*) is a flowering plant whose active chemical (mangiferin, 50 mg/kg) and extract (50, 100, or 250 mg/kg) have been shown by Rivera et al. to decrease Th2 cytokines and inflammation in the airways in the mouse allergic asthma model via reducing IgE, IL-4, and IL-5 in the serum and BALF in comparison to dexamethasone [106].

#### 3.1.34. *Mentha longifolia* (Mint or Menthol)

*Mentha longifolia* is a genus *Mentha* (mint) species. Antiasthmatic [211] and anticough [212] effects have been reported. It also reduces iNOS levels and TNF-*α* expression in lipopolysaccharide-stimulated macrophages [213].

#### 3.1.35. *Mentha piperita*

This plant has dry and warm nature and numerous medical features. The green-formulated gold nanoparticles of *Mentha piperita* possess immunomodulatory and anti-inflammatory properties, as described by Yi et al. in their study on pathological lung changes and ovalbumin-induced asthma in rats [214].

#### 3.1.36. *Moringa oleifera* Lam

This is a drought-resistant species native to India. Souresh et al. showed that *Moringa oleifera* has antiasthmatic activity and could improve the tidal volume and reduce histamine concentration and total WBC [110]. A clinical study showed an appreciable decrease in asthma symptoms and improvement in pulmonary function tests following *Moringa oleifera* administration [46]. Mahajan et al. revealed that the 100 mg/kg n-butanol fraction of the seeds inhibit airway inflammation in the guinea pig asthmatic model induced by ovalbumin, which was comparable with dexamethasone (2.5 mg/kg) [215].

#### 3.1.37. *Nigella sativa*

*Nigella sativa* is commonly known as black seed [216]. Clinical studies revealed that the *Nigella sativa* extract at concentrations of 50 and 100 mg/kg causes a significant increase in pulmonary function tests (PFTs) in most time intervals, at lower doses than theophylline (6 mg/kg) [217]. In animal models, mechanisms such as the alleviation of imbalance between the Th1 and Th2 cytokines [115] and inhibition of histamine release from mast cells [116] have been shown. Boskabady et al. showed that asthma symptoms such as night wheezing and coughing and exercise wheezing and coughing in patients improve after three months' treatment with *Nigella sativa* seed extract. Pulmonary function tests improve after 45 days of treatment [117, 218]. The antiasthmatic effects of *Nigella sativa* and its constituents have been reviewed in more detail [218]. Khaldi et al. showed that *Nigella sativa* protected against cytotoxicity and oxidative stress in a Wistar rat allergic asthmatic model induced by smokeless tobacco [219].

#### 3.1.38. *Nasturtium officinale*

*Nasturtium officinale*, known as watercress, is an aquatic plant from the cabbage family (*Brassicaceae*). It has been shown that oxidative stress and lung inflammation are reduced by 500 mg/kg *Nasturtium officinale* hydroalcoholic extract in a model of rat asthma induced by ovalbumin [220].

#### 3.1.39. *Ocimum basilicum* (Basil)

*Ocimum basilicum* is a genus of aromatic annual and perennial herbs and shrubs in the family *Lamiaceae* [118]. Eftekhar et al. demonstrated that 0.75, 1.5, and 3 mg/kg *O. basilicum* extract had immunomodulatory and anti-inflammatory effects by raising the IFN-*γ*/IL-4 ratio and improving the pathological changes in asthmatic rats compared to dexamethasone (1.25 *μ*g/kg) [118]. The results of Boskabady et al. confirmed that *O. basilicum* extract and rosmarinic acid, its constituent, reduced total and differential WBC count and serum MDA, NO_3_, and NO_2_, in sensitized rats, comparable or even stronger than dexamethasone [119].

#### 3.1.40. *Phlomis umbrosa* Turczaninow

*Phlomis umbrosa* Turczaninow is a traditional herbal medicine whose administration at concentrations of 20 and 40 mg/kg effectively reduces allergic responses in asthmatic mice compared to montelukast (30 mg/kg). This effect is associated with suppressing the phosphorylation of ERK and p65 and the expression of MMP-9 [221].

#### 3.1.41. *Phyllanthus amarus*

*Phyllanthus amarus* is widely spread throughout tropical and subtropical countries, including India (299). Wu et al. showed that the immunoinflammatory response is improved by concentrations of 50, 100 and 200 mg/kg *Phyllanthus amarus* hypophyllanthin and phyllanthin in OVA-induced asthmavia reducing TNF-*α*, IL-6, IL-4, IL-1*β*, TGF-*β*, iNOS, IgE, and Nrf2 in comparison to montelukast (10 mg/kg) [120]. Iyekowa et al. demonstrated the antiasthmatic effect of *Phyllanthus amarus* hexane extract in guinea pigs [121]. Also, the leaf extract's antiasthmatic action was demonstrated in rats compared to ketotifen fumarate [222].

#### 3.1.42. *Physalis angulata* Linn

Traditional medicine utilizes *P. angulata* to treat malaria, inflammatory diseases, and asthma. The alcoholic root extract of *P. angulata* was investigated for its antiasthmatic activity in asthmatic albino mice. The extract reduced the release of inflammatory mediators, preventing OVA-induced asthma [30, 223].

#### 3.1.43. *Pimpinella anisum*

*Pimpinella anisum* (anise), also called aniseed or rarely anix, is a flowering plant in the family *Apiaceae*. Several studies have reported the bronchodilator effects of the essential oil and alcoholic and aqueous extracts of *P. anisum*. The essential oils reduce proinflammatory cytokines, stimulating the secretion of mucus in the epithelial cell lines of the trachea and bronchi through diminishing IL-8 and IL-1*β* levels [224] and relaxes the guinea pig's tracheal chain [50, 122]. Dargahi et al. showed the anti-inflammatory effect of *Pimpinella anisum* extract in a mouse model of allergic asthma [225].

#### 3.1.44. *Pistacia atlantica*

*Pistacia atlantica* is a pistachio tree species known in English as the Persian turpentine tree and the Mount Atlas mastic tree [226]. Shakarami et al. showed the therapeutic and protective impact of 100, 200, and 400 mg/kg aqueous extract of *Pistacia atlantica* gum on the pathological and cellular qualities of experimental Balb/c asthmatic mice in comparison to dexamethasone (1 mg/kg) [123]. Also, Lee et al. revealed the protective role of *Pistacia weinmannifolia* root on lung inflammation in the mouse allergic asthma model induced by ovalbumin [227].

#### 3.1.45. *Portulaca oleracea* (Purslane)

*Portulaca oleracea* (common purslane, also known as duckweed, little hogweed, or parsley) is an annual succulent in the family *Portulacaceae*. Boskabady et al. revealed the anti-inflammatory and immunomodulatory effects of the *P. oleracea* extract at concentrations of 1, 2, and 4 mg/ml in a rat asthma model. Its reduction of the BALF levels of PLA2, TP, and IgE suggests that the plant extract can have therapeutic effects on asthma, comparable with 1.25 *μ*g/ml dexamethasone [124].

#### 3.1.46. *Punica granatum*

Pomegranate (*Punica granatum* L.) is an edible cultivar that belongs to the *Punicaceae* (now in *Lythraceae*) family [228]. Shaban et al. showed that a newly developed *Punica granatum* seed oil organogel was able to inhibit NF-*κ*B, WNT/*β*-catenin, IL-13, and oxidative stress in ovalbumin-sensitized rats compared to dexamethasone (1 mg/kg) [229, 230].

#### 3.1.47. *Sambucus nigra* (Elder)

Concentrations of 10 to 200 mg/kg of this species of the *Adoxaceae* family, found in North America and Europe, have been demonstrated by Alrumaihi et al. to substantially reduce IgE, IL-13, IL-5, and IL-4 levels and decrease BALF inflammatory cells in OVA-exposed mice. Likewise, this treatment decreased alveoli congestion and decreased the lung's inflammatory cell infiltration [136].

#### 3.1.48. *Sesamum indicum* (Sesame)

Benne or sesame is a flowering plant in the genus *Sesamum*. Hsu et al. found that sesame oil at concentrations of 0.25, 0.5, and 1 mL/kg attenuates OVA-induced bronchial neutrophilic inflammation and pulmonary edema in mice through reducing inflammatory cell count, IL-6, IL-1*β* concentration, tissue nitrite level, and serum IgE [137].

#### 3.1.49. *Sophora flavescens*

Matrine, isolated from *Sophora flavescens*, of the genus *Sophora* (*Fabaceae* family) also known as the shrubby sophora [138], suppresses Th2 cytokine (TNF-*α*, IL-13, IL-6, IL-5, and IL-4) and eotaxin production in asthmatic mice, attenuating eosinophil infiltration and allergic airway inflammation at concentrations of 5, 10, and 20 mg/kg [231]. Yang et al. showed that *Sophora flavescens* prevents airway tracheal ring smooth muscle contraction induced by acetylcholine in asthmatic mice [232]. Wang et al. showed that a *Sophora flavescens* derivative, sophoraflavanone G, inhibits oxidative stress and Th2 response in a murine asthma model, improving inflammation in allergic airways [233].

#### 3.1.50. *Glycine max* (Soybean)

Soybean or soya bean is a species of legume native to East Asia, widely grown for its edible bean. Cho et al. reported that treatment with soy isoflavone significantly reduced severe asthma flareups in asthmatic patients with high PAI-1-producing genotypes partly by decreasing the generation of PAI-1 in airway epithelial cells using genistein [234]. Bao et al. demonstrated that soy isoflavone successfully decreased AHR, airway remodeling, mucus hypersecretion, MMP-9 expression in the lung tissues, pulmonary eosinophilia, pathologic oxidizing process, and Th2 cytokine production induced by OVA in a mouse asthma model [235]. Another research demonstrated that dietary intake of soy genistein is connected to asthma control and improved lung function tests in asthmatic patients [236].

#### 3.1.51. *Thymus vulgaris* (Thyme)

This herbaceous shrub is a permanent plant from the *Lamiaceae* family [139]. Al-Khalaf et al. showed that in asthmatic mice, thyme and thymol reduce NO, hydrogen peroxide, MDA, isoprostane, and the carbonyl group and increased GPX and SOD [139]. Zhou et al. showed that 4, 8, and 16 mg/kg thymol improves the inflammation of allergic airways and AHR by reducing inflammatory cells, Th2 cytokines (IL-4, IL-5, and IL-13), and OVA-specific IgE in comparison to dexamethasone (2 mg/kg), probably associated with blockade of NF-*κ*B activation [140].

#### 3.1.52. *Tridax procumbens*

High anti-inflammatory activity and active phytoconstituent content make this plant interesting to traditional medicine. Devi et al. demonstrated that *Tridax procumbens* alcoholic extract improved pulmonary inflammation in an allergic asthma model by the inhibition of NF-*κ*B/p65/ERK-mediated signaling [237].

#### 3.1.53. *Urtica dioica*

This is a species from the *Urticaceae* family. Zemmouri et al. showed that 1.5 g/kg *Urtica dioica*, known as nettle leaf, stinging nettle, or common nettle, attenuates lung tissue lipid peroxidation and inflammation induced by ovalbumin in a rat asthma model via diminishing inflammatory cells, IL-4, and MDA and increasing GPX, SOD, and GSH in serum and BALF [141].

#### 3.1.54. *Valeriana officinalis*

This plant, known as valerian, is found in many temperate areas of America, Europe, and Asia. Its hydroalcoholic extract was shown by Neamati et al. to reduce depression-like behavior in asthmatic rats sensitized by OVA [238].

#### 3.1.55. *Viola odorata*

This native plant of Asia and Europe is a flowering member of the viola family [239]. In a clinical trial, Qasemzadeh et al. showed that viola syrup (as an adjuvant *t* of short-acting *β*-agonist) effectively reduces and suppresses intermittent asthma and cough in 2–12-year-old children [240].

#### 3.1.56. *Vitex negundo* Linn

A valuable shrub found in India, in the 1500 m altitude of Western Himalayas, *Vitex negundo* Linn. is used in folk medicine to treat respiratory disorders. Tirpude et al. found that *Vitex negundo* Linn. extract at the concentration of 100 *μ*g/ml activates macrophages and modulates the TGF-*β*/Smad/Bcl2/caspase/LC3 and AMPK/PI3K/Akt/p38-NF-*κ*B cascades, improving lung injury and inflammatory aggravation in a murine allergic asthma model induced by OVA-LPS [241].

#### 3.1.57. *Vitis vinifera*

The common grape vine is native to Southwestern Asia (East to Northern Iran), Central Europe, and the Mediterranean region. Arora et al. demonstrated that the dried fruits of *Vitis vinifera* have antiasthmatic effects at concentrations of 31 and 42.5 mg/kg compared to dexamethasone (2.5 mg/kg) in an animal model of bronchial asthma. *Vitis vinifera* reduced respiratory rate, improved tidal volume, and diminished inflammatory cells and interleukins LTD-4, IgE, NO, nitrite, and histamine levels in the serum and BALF [144].

#### 3.1.58. *Zingiber officinale* (Ginger)

The rhizome of this plant, known as ginger root or ginger is commonly employed in folk medicine and as a spice. Yocum et al. showed that ginger and its bioactive component 6-shogaol at a concentration of 6.6 mM improve lung inflammation in an asthmatic mouse model [242]. Townsend et al. demonstrated that ginger and its constituents have a bronchorelaxant effect via cell calcium regulation [145]. Another research displayed the immunomodulatory role of ginger ethanolic and aqueous extracts exerted through decreasing the mRNA expression and protein levels of Th2 cytokines in asthmatic mice compared to methylprednisolone (5 mg/kg) [146]. Also, Zhu et al. demonstrated the protective role of zingerone in a murine asthma model via activation of the AMPK/Nrf2/HO-1 pathway [243]. Jedli et al. showed the ameliorative function of *Zingiber officinale* on asthma via an increase in antioxidant potential and modulation of the STAT6 and TNF-*α* pathways [244].

### 3.2. Plants with Potentially Antiasthmatic Effects

There are herbs whose effects on asthma have not been directly studied, but due to their anti-inflammatory, antioxidative, and bronchodilatory effects, they may have antiasthmatic effects to be investigated in future studies. We found 32 of these plants in our literature review. To reduce the length of this review, the studies related to these plants are described in the supplementary file.

### 3.3. Herbal Bioactive Compounds with Antiasthmatic Effects

Researchers have tried isolating the active ingredients from the plants, showing antiasthmatic activity. In the following section, we have reviewed the literature concerning the effect of these herbal compounds on asthmatic animal models or their use in clinical trials.

#### 3.3.1. Baicalin


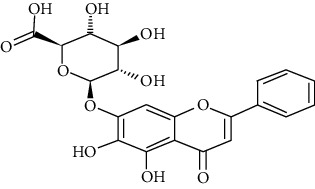


Baicalin is a flavone glycoside and a natural product from traditional Chinese medication. Xu et al. revealed baicalin at concentrations of 10, 25, and 65 mg/kg decreases OVA + LPS-induced organ coefficient (organ weight (mg)/body weight (g)) of lungs, inhibits IL-6 and IL-17A in BALF and IgE in the serum. It also alleviates airway mucus secretion and lung tissue inflammatory responses and increases IL-10 in BALF in allergic asthma induced by OVA + LPS in mice. Moreover, baicalin increases FOXP3 expression and inhibits STAT3 expression in the mouse lung tissues comparable with 1 mg/kg budesonide [55]. Liu et al. showed that 10, 25, and 50 mg/kg baicalin attenuates inflammation in mice with OVA-induced asthma by inhibiting NF-*κ*B and suppressing CCR7/CCL19/CCL21 in comparison to dexamethasone (0.085 mg/kg) [56]. Also, another study showed that baicalin mediates the TLR4/NF-*κ*B pathway and upregulates microRNA-103, regulating asthma development in children [57]. Hu et al. found that 50, 100, and 200 mg/kg baicalin modulates the RAS signaling pathway, reducing the proliferation of airway smooth muscle cells in murine asthmatic airways in comparison with dexamethasone (1 mg) [245].

#### 3.3.2. Berberine


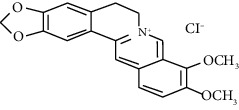


Berberine is a quaternary ammonium salt from the protoberberine group of benzylisoquinoline alkaloids found in *Berberis vulgaris* (barberry) and *Berberis aristata* (turmeric tree). Saadat et al. reported the potent relaxant effect of berberine on tracheal smooth muscles through histamine (H1) receptor blockade and inhibition of COX pathways and NO formation [58]. It has been shown that berberine regulates MAPK signaling and Fc*ɛ*RI mediation, reducing allergic responses mediated by mast cells [59]. One study found that 0.1, 1, and 10 *μ*M berberine modulates the STAT6 pathway in bronchial epithelial cells in humans, suppressing the production of proinflammatory CCL11 and IL-6 induced by cytokines [60].

#### 3.3.3. Carvacrol


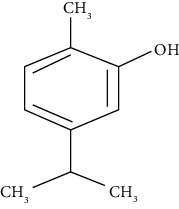


Carvacrol is a monoterpenoid phenol present in the essential oil of *Origanum vulgare* (oregano), oil of thyme, oil of pepperwort, and wild bergamot [246]. Khalaf et al. demonstrated that at a concentration of 15 mg/kg, carvacrol diminishes inflammation in bronchial asthma via reducing tissue MDA and BALF TNF-*α*, IFN-*γ*, and iNOS levels and increasing SOD and GSH compared to dexamethasone (1 mg/kg) [66]. Boskabady et al. showed a preventive effect of carvacrol, as potent as dexamethasone, on tracheal responsiveness at 40, 80, and 160 *μ*g/mL [67]. Also, they reported that carvacrol attenuates serum levels of total protein, phospholipase A2, and histamine in asthmatic guinea pigs [247]. Some clinical investigations have shown improvement in respiratory symptoms, pulmonary function tests, and FEV1 values and alleviation of lung wheezing in asthmatic patients following treatment for two months. Also, in a randomized, double-masked clinical trial, carvacrol improved pulmonary function tests, oxidant/antioxidant parameters, and cytokine levels in asthmatic patients [34].

#### 3.3.4. Ellagic Acid


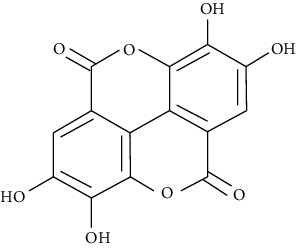


A phenol antioxidant naturally occurring in various vegetables and fruits is ellagic acid. Ellagic acid has been found to possess antioxidant and antiproliferative effects [79]. Alves et al. revealed that ellagic acid decreases eosinophil recruitment and airway mucus metaplasia and promotes the resolution of airway allergen clearance [80, 81]. Zhou et al. reported that ellagic acid at concentrations of 2.5, 5, or 10 mg/kg attenuates airway inflammation, release of Th2 cytokines into the airways, goblet cell hyperplasia, OVA-specific IgE levels in the serum, and AHR in an OVA-induced asthmatic mouse model compared to dexamethasone [248].

#### 3.3.5. Emodin


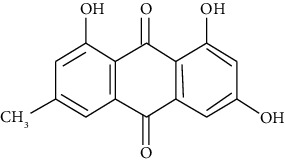


Abundant in the roots of knotgrass, knotweed, and Chinese rhubarb, emodin is a compound in the anthraquinone family. Miao et al. reported that emodin inhibits the NF-*κ*B signaling pathway, protecting obese asthmatic rats from pathological damage via visfatin [249].

#### 3.3.6. Eupatilin


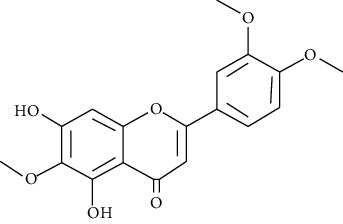


Extracted from *Artemisia argyi*, eupatilin is a pharmacologically active flavone with neuroprotective, cardioprotective, antiallergic, antioxidant, anticancer, and anti-inflammatory activities. Bai et al. demonstrated that eupatilin activates the Nrf2 signaling pathways and inhibits MAPK and NF-*κ*B, suppressing asthma induced by OVA in mice [250].

#### 3.3.7. Evodiamine


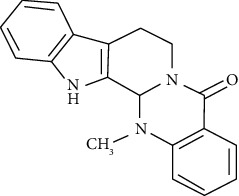


Evodiamine, isolated from *Evodia rutaecarpa* (*Rutaceae*), is a traditional medicine used in China. Wang et al. showed that at concentrations of 40 and 80 mg/kg, evodiamine protects against airway remodeling and inflammation in asthmatic rats by modulating the HMGB1/NF-jB/TLR-4 signaling pathway [251].

#### 3.3.8. Fisetin


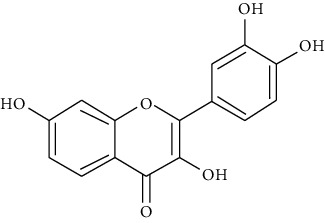


A member of flavonoid polyphenols, fisetin (7, 3′, 4′-flavon-3-ol) is a plant flavonol [82] with beneficial effects on airway hyper-reactivity through reduction of allergic inflammation and airway resistance [83]. Paul et al. showed that orally administered fisetin at the concentration of 2 *μ*M/kg in a mouse chronic allergic airway disease model reduces inflammatory cells, IgE, IL-13, IL-5, IL-4, IL-3, TGF-beta, and iNOS in BALF, serum, and tissue [84]. Goh et al. demonstrated that administration of fisetin at 0.3, 1, and 3 mg/kg effectively reduces ovalbumin-induced pulmonary eosinophilia, mucus hypersecretion, *v*, and AHR in an acute mouse asthma model. Also, they reported that fisetin diminishes IL-17, IL-33, VCAM-1, and iNOS in asthmatic mice [85]. It has been shown that acute allergic asthma symptoms are reduced following oral administration of fisetin in a preclinical mouse model [252].

#### 3.3.9. Gallic Acid


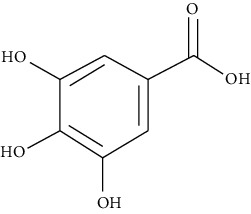


Classified as a phenolic acid, gallic acid is a trihydroxy benzoic acid [41]. Fan et al. demonstrated that oral gallic acid significantly attenuates inflammation of airways in an airway inflammation model induced by OVA. Kim et al. disclosed that gallic acid at 0.1, 1, and 10 *μ*M inhibits histamine release and proinflammatory cytokine (IL-6 and TNF-*α*) production in mast cells [253]. The effects of gallic acid are probably connected to inactivated ILC2s and suppressed release of IL-13 and IL-5 via the IL-33/MyD88/NF-*κ*B signaling pathway [254].

#### 3.3.10. Genistein


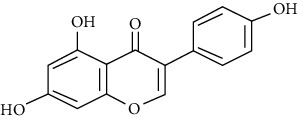


Structurally a member of isoflavone compounds, genistein occurs naturally in plants [255]. Liu et al. showed that genistein can inhibit the translocation of NF-*κ*B and hypersecretion of TNF-*α* in PBMCs of asthma patients. However, the effects were weaker than those of dexamethasone [86]. Another study revealed that genistein modulates the transcription factors STAT6, GATA3, and T-bet, attenuating airway allergic inflammation in a murine asthma model [87]. Duan et al. also showed anti-inflammatory effects for 15 mg/kg genistein in a guinea pig asthma model as a tyrosine kinase inhibitor [256].

#### 3.3.11. Hesperetin


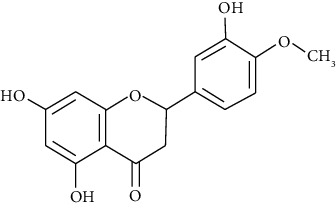


Hesperetin is a naturally occurring flavanone glycoside [94], which selectively inhibits phosphodiesterase four and effectively suppresses OVA-induced AHR [95]. In a clinical study, Gu et al. disclosed that hesperetin inhibits the maturation and function of monocyte-derived dendritic cells from patients with asthma by suppressing the activation of NF-*κ*B, reducing IL-4, increasing IFN-*γ* levels [45], ameliorating airway inflammation, remodeling, and decreasing airway fibrosis and mucus plug formation [257]. AHR Alleviation, BALF Th2 cytokine level enhancement, inflammatory cell reduction, goblet cell hyperplasia attenuation, and airway luminal narrowing are the other antiasthmatic mechanisms of hesperetin [99].

#### 3.3.12. Iristectorigenin A


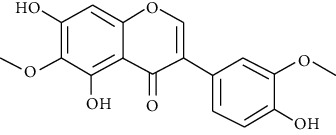


Iristectorigenin A, a naturally occurring isoflavone, is extracted from various medical plants. Novel protective properties have been shown for iristectorigenin A against hypersecretion of mucus and inflammation of airways in asthma induced by OVA in mice [258].

#### 3.3.13. Jiawei Shengjiang

Jiawei Shengjiang powder is a classical Chinese medicinal formula widely applied for many years in treating asthma and its complications. Wang et al. demonstrated that Jiawei Shengjiang powder at the concentration of 1.59 g/kg improves asthma consequences by reducing IL-6 and TNF-*α* [259].

#### 3.3.14. Kaempferol


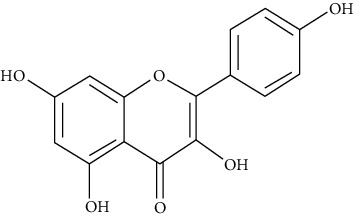


Kaempferol is a natural flavonol found in various plants and plant-derived foods, including kale, beans, tea, spinach, and broccoli [260]. Molitorisova et al. revealed that long-term use of kaempferol at concentrations of 2, 6, or 20 mg/kg attenuates the progression of chronic inflammation by decreasing the amount of proinflammatory cytokines IL-5, IL-13, GM-CSF, and eosinophils in the BALF compared to budesonide [101]. In addition, kaempferol attenuates TNF-*α*-induced expression of epithelial ICAM-1, integrin *β*2, and MCP-1 transcription and ameliorates allergic and inflammatory airway diseases [261]. Medeiros et al. showed that kaempferol treatment (both preventive or curative) exerts a profound inhibitory effect on airway inflammation and hyperresponsiveness in a murine asthmatic model by suppressing the Th2 cytokine profile via reducing IL-5 and IL-13 [262]. Xu et al. showed that kaempferol (20 mg/kg) inhibits airway inflammation induced by allergic asthma through NOX4-mediated autophagy [263].

#### 3.3.15. Kaurenoic Acid


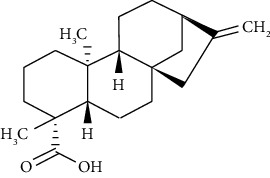


Kaurenoic acid is a diterpene from many plants, such as *Sphagneticola trilobata* [264]. Borghi et al. demonstrated that 10 mg/kg kaurenoic acid prevents ovalbumin-induced asthma in mice through its effect on Th2 cytokines, STAT6/GATA3 signaling, NF-*κ*B/Nrf2 redox-sensitive pathways, and regulatory T cell phenotype markers [265].

#### 3.3.16. Kanakasava

Kanakasava is one of the polyherbal formulations widely used for the treatment of allergic conditions like chronic cough, bronchial asthma, and breathing disorders. Arora et al. demonstrated how Kanakasava exerts its antiasthmatic effect at concentrations of 1.23 and 2.46 ml/kg in ovalbumin-induced airway inflammation and bronchial asthma in rats by reducing IL-5, IL-4, TNF-*α*, IL-1*β*, LTD-4, IgE, NO, and nitrite in serum and BALF compared to dexamethasone [103].

#### 3.3.17. Ligustrazine


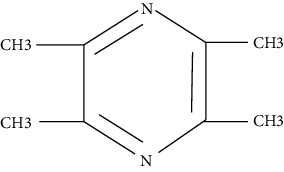


Ligustrazine is an alkaloid derivative of the Chuanxiong rhizome (*Ligusticum chuanxiong Hort*). Wang et al. disclosed that in an asthma model, inflammation and hyperresponsivity induced by allergens in the lungs is reduced significantly by ligustrazine. Treatment with ligustrazine has been connected to decreased PDE expression, including the expression of PDE4, in the rat lungs [266].

#### 3.3.18. Limonene


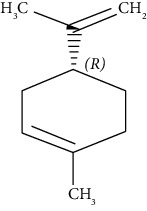


Limonene is a colorless liquid aliphatic hydrocarbon, classified as a cyclic monoterpene, a significant component in the oil of citrus fruit peels. Rajalingam et al. reported that limonene reduces tracheal and vascular reactivity in a mouse asthmatic model [267]. Patel et al. revealed that limonene-induced activation of A2AARs reduces airway inflammation and reactivity in a mouse asthmatic model via reducing inflammatory cells and IgE in the BALF [268].

#### 3.3.19. Morin


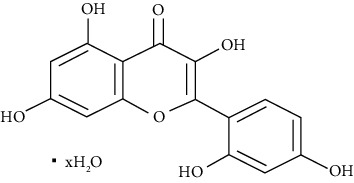


Common guava (*Psidium guajava*) leaves, old fustic (*Maclura tinctorial*), and Osage orange (*Maclura pomifera*) can be used to isolate the yellow compound called morin [269]. Franova et al. revealed morin's bronchodilator, anti-inflammatory, and antitussive effects at 30 mg/kg in experimentally induced allergic asthma via reducing airway resistance, number of cough efforts, and the concentration of IL-4, IL-5, and IL-13 in the BALF of asthmatic guinea pigs compared to dexamethasone [107]. Kandhare et al. found that morin possesses antiasthmatic qualities mediated by the reduction of oxidative airway inflammation and stress through modulating the BLT2/NF-*κ*B and SUMF2/IL-13 signaling pathways and downregulating the expression of LTB-4, IL-6, IL-4, COX-2, and IgE [108] in OVA-induced allergic asthma [270].

#### 3.3.20. Myrtenol


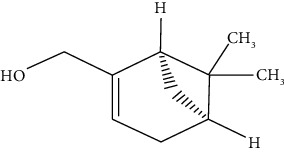


True myrtle or the common myrtle, is a flowering member of the *Myrtaceae* family [271–273]. Bejeshk et al. demonstrated that myrtenol exerts antiremodeling effects in asthmatic rats' lungs by reducing the remodeling process in the airways. Also, they showed myrtenol's oxidation- and inflammation-reducing effects at 50 mg/kg through diminishing MDA, TNF, and IL-1*β* and increasing BALF SOD, GPX, IFN, and IL-10 [111]. In another study, Rajizadeh et al. revealed that myrtenol at 50 mg/kg can reduce inflammation and tissue damage in asthmatic rats compared to dexamethasone (2.5 mg/kg) [112]. Our recent investigations revealed the oxidation- and inflammation-reducing effects of the inhaled niosomal form of myrtenol in asthmatic rats [274, 275].

#### 3.3.21. Narciclasine


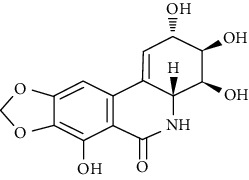


Narciclasine, an isocarbostyril alkaloid, was first isolated from *Haemanthus coccineus* L. (family: *Amaryllidaceae*). Peng et al. reported that narciclasine modulates matrix remodeling in asthmatic neonatal rats by regulating inflammatory pathways [276].

#### 3.3.22. Naringenin


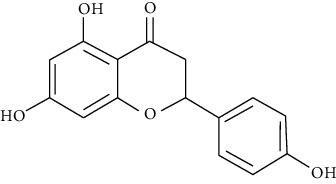


Naringenin is a colorless, flavorless flavonoid predominantly found in grapefruit and other fruits and herbs [113]. A study showed that consumption of hesperetin-naringenin is associated with substantial amelioration of airway structural changes and provided significant insight into the role of orange juice or grapefruit juice in decreasing airway fibrosis and mucus plug formation in an asthma model [257]. Shi et al. reported that naringenin at 25, 50, and 100 mg/kg inhibits allergen-induced airway inflammation by reducing serum IgE, inflammatory cells, CCL11, CCL5, IL-4, and IL-13 in BALF. It also ameliorates airway responsiveness and airway remodeling and decreases NF-*κ*B activity in asthma [114, 277]. Jasemi et al. revealed that the antioxidant and anti-inflammatory effects of naringenin improve the allergic asthma induced by ovalbumin in rats [278].

#### 3.3.23. Osthole


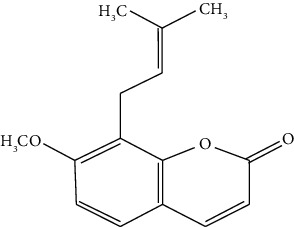


Osthole, one of the most essential coumarin compounds isolated from the ripe fruit, is derived from the plant *Cnidium monnieri* (L.) Cuss. Tong et al. disclosed the ameliorating role of osthole on TGF-*β*-induced lung epithelium apoptosis and epithelial-mesenchymal transition-mediated airway remodeling in pediatric asthma [279].

#### 3.3.24. Qingfei Yihuo Wan

Jing et al. revealed that qingfei liquid administered orally at the concentration of 6.36 g/kg alleviates AHR and mucus hypersecretion via TRPV1 signaling in RSV-infected asthmatic mice. They reported that qingfei ameliorates airway resistance and lung compliance and reduces serum levels of IL-4, IL-5, IL-13, and IL-1*β* compared to dexamethasone [129]. Yu et al. demonstrated that qingfei oral liquid inhibits autophagy via the mTOR signaling pathway in RSV-infected asthmatic mice [130]. Another study showed that long-term use of qingfei reduces the risk of asthma hospitalization in school-age children [280].

#### 3.3.25. Quercetin


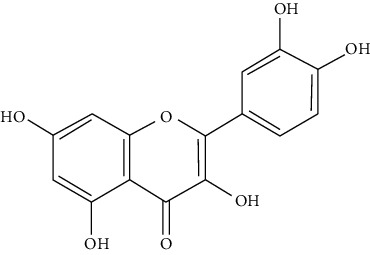


A plant flavonol, quercetin belongs to the flavonoid polyphenols group [281]. Rogerio and colleagues showed the anti-inflammatory effect of quercetin-loaded microemulsion by reduction of eosinophil recruitment and IL-4 and IL-5 levels in the BALF and P-selectin expression and mucus secretion in the lung 1 and 10 mg/kg in the airways of an allergic inflammatory model in mice in comparison to dexamethasone (1 mg/kg) [125, 282]. Park et al. revealed that quercetin decreases the expression of T-bet and suppresses GATA3, reducing hyperresponsiveness and inflammation of allergic airways and regulating the Th1/Th2 balance in a murine asthma model [126]. Another study confirmed that quercetin acts as an acute bronchodilator in experimental allergic asthma [283]. Ravikumar and Kavitha revealed the immunomodulatory effect of quercetin on dysregulated Th1/Th2 cytokine balance in mice with both type 1 diabetes and allergic asthma [127]. In another study, Sozmen et al. demonstrated how treatment with quercetin affects epithelial cell apoptosis and epithelium-derived cytokines in a mouse allergic airway inflammation model [128]. Our previous study showed quercetin's oxidation- and inflammation-reducing effects in asthmatic rats by reducing GATA3 and increasing T-bet expression [284]. Also, Fang et al. demonstrated that quercetin alleviates asthma-induced airway inflammation and remodeling through downregulating periostin via blocking the TGF‐*β*1/Smad pathway [285].

#### 3.3.26. Resveratrol


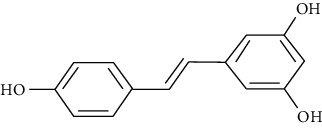


Peanuts, raspberries, blueberries, mulberries, and the skin of grapes contain resveratrol [131, 132]. Antioxidant and anti-inflammatory effects, reduction of airway remolding and TNF-*α*, TGF-*β*, IL-17, and IL-6 in the BALF of asthmatic mouse model [286], attenuation of experimental allergic asthma, restoration of mitochondrial function, inhibition of PI3K-Akt signaling, and reduced calpain activity in mice are among the antiasthmatic actions of resveratrol [133]. Moreover, the oral administration of resveratrol suppresses the asthma-associated immune response mediated by the upregulation of FOXP3 and downregulation of miR-34a [287]. Chen et al. confirmed the antiasthmatic effects of resveratrol at 30 mg/kg in a mouse OVA-induced asthma model compared to dexamethasone (5 mg/kg) [288]. Hu et al. showed the effective suppression of eosinophil proliferation by resveratrol treatment in asthmatic patients [289].

#### 3.3.27. Rosmarinic Acid


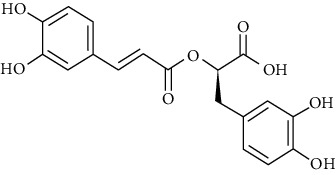


Rosmarinic acid is a compound from *Rosmarinus officinalis* L. (rosemary), which is a medicinal plant native to the Mediterranean region and cultivated around the world [135]. Shakeri et al. showed that at concentrations of 0.125, 0.25, and 0.50 mg/ml (1.25 *μ*g/ml), similar to dexamethasone, rosmarinic acid improves immunological and inflammatory mediator levels by reducing IL-4, PLA2, TP, and IgE and increasing IFN-*γ* in BALF and ameliorates lung pathological insults in asthmatic rats [290]. Liang et al. revealed that rosmarinic acid treatment at the concentration of 20 mg/kg results in a significant reduction in the mRNA expression of AMCase, CCL11, CCR3, Ym2, and E-selectin in the lung tissue of asthmatic rats [291]. Our previous study revealed that the inhalation of *Salvia rosmarinus* Spenn. has oxidation- and inflammation-reducing effects in asthmatic rats [292].

#### 3.3.28. Ruscogenin


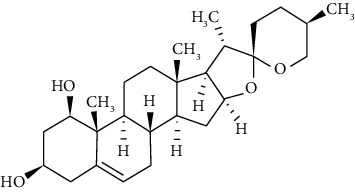


Ruscogenin is one of the major active compounds in *Ophiopogon japonicus*, which has been shown to exert anti-inflammatory, antioxidant, and antiapoptotic effects. Zhan et al. demonstrated that 10 mg/kg ruscogenin inhibits mitochondrial calcium handling and VDAC1 expression, reducing apoptosis and oxidative stress in the airway epithelium [293].

#### 3.3.29. Syringin


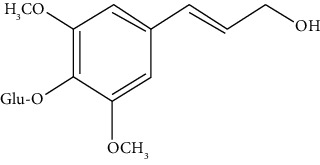


Eleutheroside B or syringin is a phenylpropanoid glycoside extracted from *Eleutherococcus senticosus*, *Jasminum mesnyi*, *Edgeworthia chrysantha Lindl.*, and *Acanthopanax senticosus* [294]. Dai et al. revealed that syringin uses the NF-*κ*B signaling pathway to reduce inflammation of the lungs induced by ovalbumin in a BALB/c mouse asthmatic model [295].

#### 3.3.30. Tyrosol


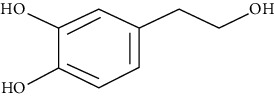


Tyrosol is a phenolic compound found mainly in olive oil and white wine. Cellat et al. disclosed that 20 mg/kg tyrosol alleviates OVA-induced asthmatic rat model by preventing inflammation of the airways by reducing IgE, IFN-*γ*, NF-*κ*B, TNF-*α*, IL-4, IL-13, and IL-5 levels in the lung tissue and serum comparable to dexamethasone (0.25 mg/kg) [296].

#### 3.3.31. Vanillic Acid


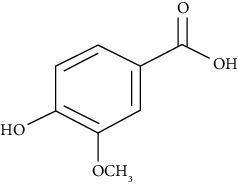


Vanillic acid is a derivative of dihydroxybenzoic acid employed for flavoring. Bai et al. showed that vanillic acid at concentrations of 25 and 50 mg/kg mitigates airway inflammation in the OVA-induced rat asthma model via reducing inflammatory cells and TNF-*α*, IL-13, IL-5, and IL-4 levels and improving oxidative stress compared to dexamethasone (3 mg/kg) [142]. Jeong et al. reported that by controlling the activated mast cell secretion of thymic stromal lymphopoietin, vanillic acid exerts its antiallergic effect [297].

#### 3.3.32. Vitexin


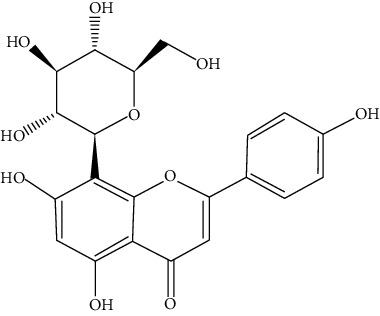


Found in the bamboo leaves (*Phyllostachys nigra*) and passion flower (*Vitex agnus-castus*, chasteberry), vitexin is an apigenin flavone glycoside [298]. Venturini et al. showed that vitexin at concentrations of 0.2, 1, and 5 mg/kg inhibits inflammation in a murine model of OVA-induced allergic asthma via reducing IgE in the serum and inflammatory cells and IL-5, IL-4, and IL-13 in BALF, comparable to dexamethasone (0.5 mg/kg) [143]. Also, modulation of the PTEN, GATA3, p-Stat3, p-p38, p-Akt, p-PI3K, p-NF-*κ*B, BAX/Bcl2, LC3A/B, caspase 9/3, Smad2/3/4, and TGF-*β* pathways by vitexin has been shown in an asthmatic mice model [299].

## 4. Discussion

**Figure 2:**
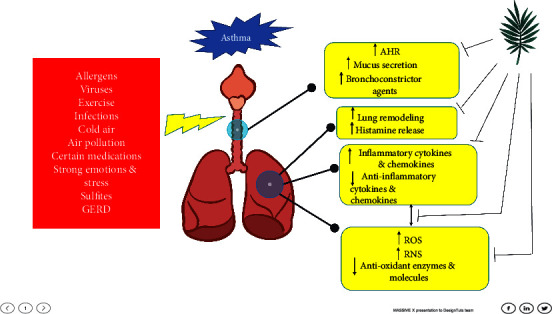
Factors involved in asthma induction (left) and the signs and symptoms of disease (middle), along with the mechanisms of antiasthmatic effects of plants or their compounds (right). GERD (gastroesophageal reflux disease), AHR (airway hyperresponsiveness), ROS (reactive oxygen species), and RNS (reactive nitrogen species).

In this comprehensive review, we conducted a survey of 37 years of literature on herbs and their compounds used in the treatment of asthma. We found more than a hundred and twenty plants and herbal derivatives that were either beneficial for treating asthma or could potentially treat asthma. The main mechanism of action in these plants is their anti-inflammatory activity. Nevertheless, other important antiasthmatic mechanisms include antioxidant, antihistaminic, and antiallergic activities, inhibition of airway remodeling, and tracheal smooth muscle relaxation (see Figure 2). These results will help researchers isolate and identify the phytochemicals with antiasthmatic properties in these plants for the development of novel antiasthmatic medications. The bioactive ingredients' synergistic and additive qualities are also a possible research field for producing more effective compound formulas.

We acknowledge some limitations in our work. This review mostly looks at the mechanism of action of various plants or herbal compounds against asthma. To avoid lengthening the text, because of the large number of items found, we did not include detailed information about the route of administration, the part of the plant used, and the method of preparing the extracts. The readers may refer to the references cited for each case for more detailed information if they are interested. Also, because of the plant's anti-inflammatory, antioxidative, and bronchodilatory properties that may have antiasthmatic effects, they are described in a supplementary file. Moreover, in cases where more than one paper reported an individual property of a plant (for example, the anti-inflammatory effect), only the more recent/more comprehensive study was cited.

Most clinical trials resulted in ameliorated respiration and led to desirable outcomes (i.e., decrease in severity and frequency of symptoms) following herbal medicine administration in asthma treatment. However, due to the limited number and reliability of findings, this review calls for further studies to identify the antiasthmatic effect and the suitable dosage for relieving the disease symptoms. At this time, given the limited scientific evidence, we cannot confidently decide how effective the plant-based medications are in asthma treatment. Nevertheless, these treatment options are worth considering. High-quality studies on the long-term safety and efficacy of phytochemicals used for asthma treatment are still necessary. Furthermore, the unknown consequences of combining herbal medicine with other undeclared chemicals, toxic contamination of herbs, the use of the wrong plant species, and misuse by healthcare providers or consumers are among the reasons for experiencing adverse events arising from the consumption of herbal medicines [300].

Since the pharmacokinetic and pharmacodynamic characteristics of most herbal and other dietary supplements are only partially recognized, potential interactions are often not avoidable. Potential interactions are likely to occur with drugs with narrow therapeutic indices. Despite these warnings, almost all studies that have investigated the effects of herbal medicine on asthma have reported desirable effects. Therefore, the results of this field are promising.

## 5. Conclusion

Among all mentioned plants and compounds that have verified ameliorative effects on asthma, the main mechanism of action of these herbs is their anti-inflammatory effects. The inhibition of airway remodeling, relaxation of tracheal smooth muscles, antihistaminic properties, alleviation of airway hyperresponsiveness, and antioxidant activities are also critical antiasthmatic mechanisms of action. These results can be helpful for the researchers to identify phytochemicals present in each plant or familiarize themselves with herbal compounds to develop new antiasthmatic drugs. Along with investigating the safety and adverse effects of the mentioned plants and compounds, assessing the additive/synergistic effects of combinations of different bioactive ingredients for the production of more effective formulas with fewer side effects can become the future fields of research in order to pave the path of using herbal treatments in the clinic.

### 5.1. Practical Recommendations for Healthcare Practitioners

Based on the findings of this review, healthcare practitioners are advised to pay attention to herbal and traditional medicine to treat asthma with a positive outlook. Since herbal medicine has fewer side effects and is cheaper than the existing drugs, prescribing herbal medicine to asthma patients can help the economics of treatment. However, it is recommended that possible toxicities (e.g., in the nerves, liver, and kidneys) and drug interactions be carefully considered before prescribing herbs and herbal compounds to treat patients.

## Figures and Tables

**Table 1:** The plants or herbal compounds that have shown antiasthmatic effects in clinical studies and their mechanism of action.

Plants or compound	Effect on airways	Effect on pulmonary function tests	Reference(s)
*No.3.1.6 Argemone mexicana*	Inhibiting bronchospasm		[27]
*No.3.3.1 Baicalin*	↓ AHR, improving airway resistance and lung compliance		[28, 29]
*No.3.1.8 Biophytum sensitivum*	Inhibiting histamine-induced bronchoconstriction		[30]
*No.3.1.9 Boswellia serrata*		↑ FEV1, FVC, PEFR ↓ Episodes of asthmatic attacks	[31, 32]
*No.3.1.13 Carum copticum*	Bronchodilator effect	↑ FEV1, PEF, MMEF, MEF75, MEF50, MEF25, and sGaw	[33]
*No.3.3.3 Carvacrol*	↓ AHR	↑FEV1, FVC, MMEF, PEF, MEF75, MEF50, and MEF25	[34–36]
*No.3.1.15 Crataegus pinnatifida*	↓ AHR		[37]
*No.3.1.16 Crocus sativus*	↓ AHR		[38, 39]
*No.3.1.17 Curcuma longa rhizome (curcumin)*	↓ AHR		[40]
*No.3.3.9 Gallic acid*	↓ AHR		[41]
*No.3.1.25 Ginkgo biloba*		Improved FEV1/FVC	[42]
*No.3.1.26 Glycirizine glabra*	↓ AHR	Improved FVC and FEV1, ↓ PIF, PEF, TV, and EV	[43]
*No.3.1.27 Hedera helix*	↓ Airway resistance		[44]
*No.3.3.11 Hesperetin*	↓ AHR		[45]
*No.3.1.36 Moringa oleifera*		Improvement of FVC, FEV1, FEV1/FVC, PEFR, FEF 25–75%, and MVV	[46]
*No.3.3.22 Naringenine*	↓ AHR		[47]
*No.3.1.37 Nigella sativa*		↑ FVC, FEV1, MMEF, MEF50, MEF75, and MEF25	[48, 49]
*No.3.2.43 Pimpinella anisum*	Bronchodilator activity		[50]

The number in the first column refers to the paragraph in the text describing the plant/compound characteristics.

**Table 2:** Plants or herbal compounds with verified preclinical (animal/organ/tissue) antiasthmatic effects and related mechanisms.

Plant or compound	Anti-inflammatory	Antioxidative and antihistaminergic	References
*No.3.1.1 Adiantum capillus-veneris L. (fern)*		H1 receptor antagonist	[51]

*No.3.1.2 Allium cepa (onion)*	↓ BALF IgE, IL-4, IL-5, IL-13 ↑ BALF IFN-*γ* & IFN-*γ*/IL-4 ratio	↓BALF NO2, NO3, MDA ↑BALF SOD, CAT, thiol	[52, 53]

*No.3.1.6 Argemone mexicana*	↓ Eosinophil count		[27]

*No.3.1.7 Artemisia pallens*	↓ Serum IgE and lung TNF-*α*, IL-6, IL-4, IL-1*β*, TGF-*β* expression ↑ Lung Nrf2 expression	↓ BALF MDA, MPO, NO ↑ BALF SOD, GSH	[54]

*No.3.3.1 Baicalin*	↓ Serum and BALF IgE and BALF IL-17A and IL-6 and lung STAT3 expression ↑ BALF IL-10 and lung FOXP3 expression ↓ Eosinophil count NF-*κ*B and TLR4 pathways inhibition CCR7/CCL19/CCL21 suppression ↑ miR-103 upregulation		[28, 29, 55–57]

*No.3.3.2 Berberine*	Inhibition of COX pathways mast cell-mediated allergic responses suppression FcɛRI-mediated and MAPK signaling regulation ↓ IL-4, TNF-*α*, IL-5, IL-6, IL-13, IL-17, IL-1*β* ↓ IgE NF-*κ*B signaling pathway inhibition ↓ CCL11 production STAT6 pathway modulation	↓ NO formation H1 receptors blockade	[58–61]

*No.3.1.8 Biophytum sensitivum (L.)*		↓ Histamine release	[30]

*No.3.1.9 Boswellia serrata (Frankincense)*	↓ Plasma level of LTC4 ↓ Eosinophil count ↓ ESR ↓ Serum IL-4, IL-5, and IL-13 ↓ OVA-specific IgE ↓ Lung pSTAT6 and GATA3 expression	↓ Plasma level of MDA and NO	[31, 32, 62]

*No.3.1.10 Brassica napus (rapeseed)*	↓ Lung eosinophil count		[63]

*No.3.1.11 Camellia japonica*	GATA3 and IL-4 pathways suppression ↓ BALF IgE, eosinophil number, and WBC ↓ Lung IL-4, IL-5, IL-6, IL-13, and TNF-*α*		[64]

*No.3.1.12 Camellia sinensis (tea)*	↑ Lung TNF-*β*, IFN-*γ*, and IL-10 expression ↓ Lung IL-4 and IL-13 expression ↓ Lung IgE		[65]

*No.3.3.3 Carvacrol*	↓ Lung TNF-*α* ↓ Total and differential WBC in the blood and hs-CRP ↓ Serum levels of TP and PLA2 ↓ Serum IL-4 ↑ Serum IFN-*γ* ↓ Serum endothelin ↑ Lung FOXP3 expression ↓ Lung IL-4, IL-17, and TGF-*β* expression	↓ Lung MDA, iNOS ↑ Lung SOD, GSH ↓ Serum level of NO and nitrite ↓ Serum histamine	[36, 66–69]

*No.3.1.15 Crataegus pinnatifida*	↓ Eosinophil number in BALF and lung ↓ BALF IL-4, IL-5, IL-13, eotaxin ↓ Serum total IgE, OVA-specific IgE, OVA-specific IgG downregulation of MMP-9 ↓ ICAM-1, VCAM-1		[37]

*No.3.1.16 Crocus sativus*	↓ BALF eosinophil number ↓ Lung IL-5, IL-13, IL-4, TNF-*α*, IL-1*β* ↓ BALF IL-13, IL-4, TNF-*α*, IL-1*β* ↓ Serum IL-4 ↑ Serum IFN-*γ* ↓ Serum and BALF total WBC ↓ Serum ET-1 and TP ↓ Lung TP	↓ iNOS level ↓ NO production & nitrite ↓ Peroxynitrite ion formation ↓ Cytochrome C release H1 receptor inhibition	[38, 70–72]

*No.3.1.17 Curcuma longa rhizome (Curcumin)*	↓ Lung inflammatory cells infiltration ↓ Lung and BALF IL-4, IL-5, IL-6, IL-8, TNF-*α*, IL-17A ↓ Serum and spleen IL-4, IL-13 ↑ BALF IL-10, IFN-*γ* ↓ Serum and BALF IgE regulation of CD4+ CD25+ regulatory T cells (Tregs)/Th17 balance ↓ Serum LTC4 ↓ Spleen IL-6, IL-1*β* modulating NF-ĸB activation and MAPK signaling ↓ COX-2 ↓ P38, JNK, and ERK phosphorylation modulation of Wnt/beta-catenin signaling ↓ BALF total WBC, PLA2, TP, IgE, IL-4 modulation of Nrf2/HO-1 signaling pathway	↓ Lung iNOS expression ↓ NO expression ↓ Serum and BALF MDA, NO, NO_2_, NO_3_ ↓ Spleen iNOS ↓ ROS ↑ Serum and BALF SOD, CAT, thiol	[73–77]

*No.3.1.21 Echinodorus scaber Rataj*	↓ BALF IL-4, IL-5, IL-13, IgE		[78]

*No.3.3.4 Ellagic acid*	↓ BALF inflammatory cells ↓ BALF and lung IL-5, IL-4, IL-13, IL-33 ↓ Lung IL-6, IL-8, and CCL2 ↑ Lung IL-10 ↓ Lung TLR4, MyD88, P65, NF-*κ*B and STAT3 expression		[79–81]

*N0.3.3.8 Fisetin*	↓ Total inflammatory cells count in BALF, blood, bone marrow, spleen, lymph nodes, and thymus ↓ BALF, lung, and serum IL-3, IL-4, IL-5, IL-13, IgE, TGF-*β*, IL-17, IL-33, IL-1*β*, TNF-*α*, IL-2, IL-18 inhibition of NF-*κ*B activity modulation of MAPK, PI3K, Wnt/catenin, and ERK 1/2 pathways ↓ VCAM-1 inhibition of MyD88/NF-*κ*B signaling pathway ↓ Eotaxin ↓ BALF EPO activity	↓ BALF, lung, and Serum iNOS ↓ NO ↓ BALF histamine content	[82–85]

*No.3.3.9 Gallic acid*	↓ Eosinophil cell infiltration in the nasal mucosa ↓ NALF IL-4, IL-5, IL-13, IL-17, ROR-*γ*t ↓ Serum OVA-specific IgE and IgG ↑ NALF IFN-*γ*, IL-12 ↓ BALF IL-6, TNF-*α*, IL-5, IL-13 inhibition of IL-33/MyD88/NF-*κ*B signaling pathway	↓ Histamine release	[41]

*No.3.3.10 Genistein*	Modulation of T-bet, GATA3, and STAT6 ↓ Serum eosinophils and LTC4 ↓ 5-LO activity inhibition of p38 and MAPKAP-2 pathways		[86, 87]

*No.3.1.25 Ginkgo biloba*	↓ IgE and IL-4 ↓ BALF leukocytes and eosinophils, IL-4, IL-5, IL-13, TNF-*α*, eotaxin inhibition of the ERK/MAPK signaling pathway ↓ Serum PAF		[88–91]

*No.3.1.26 Glycirizine glabra*	↓ Eosinophils ↓ BALF IL-4, IL-5 ↑ BALF IFN-*γ* ↓ Serum and BALF IL-5, IL-13, IgE modulation of NF-*κ*B, STAT6, and HDAC2	↓ Serum and BALF MDA, NO ↑ Serum and BALF SOD, CAT	[44, 92, 93]

*No.3.3.11 Hesperetin*	Inhibit PDE4 and PDE3/4 ↓Serum and BALF IL-5, IL-4, IgE ↓ GATA3 and NF-*κ*B activity ↑ BALF IFN-*γ*		[45, 94–96]

*No.3.1.28 Hyssopus officinalis* L. *(Zufa)*	Regulation of MMP-9/TIMP-1 ratio ↓Lung IL-1*β*, TNF-*α*, IL-17 expression ↓ BALF eosinophil number and IgE ↑ BALF IgG ↓ BALF eotaxin-2, eotaxin-3, and P-selectin ↓ IL-2, IL-6, TNF-*α*, TGF-*β* ↓ Serum and BALF IL-17, IL-4, IL-6, TNF-*α* ↑ Serum IFN-*γ* regulation the expression of T-bet, GATA3, and STAT3 mRNA in lung tissue inhibiting of MAPK/NF-*κ*B pathway		[97–99]

*No.3.3.13 Jiawei shengjiang*	↓ IL-6, TNF*α*		[100]

*No.3.3.14 kaempferol*	↓ BALF IL-4, IL-5, IL-13, GM-CSF, TNF-*α*, TGF-*β*, eosinophil's count disturbing NF-*κ*B signaling ↓ Eotaxin1 ↓ ICAM-1 modulation of JAK2 signaling pathway disturbing Tyk-STAT and ERK signaling pathways ↓ COX-2, PGD 2, PGF2*α*, PLA2	↓ ROS production	[101, 102]

*No.3.3.16 Kanakasava*	↓ Inflammatory cells in blood and BALF ↓ Serum and BALF IL-4, IL-5, TNF-*α*, IL-1*β*, LTD-4, IgE	↓ Serum and BALF NO, nitrite	[103]

*No.3.1.30 Lavandula stoechas* L.	↓ Serum IgE and IgG ↓ BALF eosinophil count, IL-5, IL-13, IL-33	↓ Serum MDA ↑ Serum SOD	[104]

*No.3.1.32 Mandevilla longiflora*	↓ BALF inflammatory cells, IL-4, IL-5, IL-13, IgE, LT-B4		[105, 106]

*No.3.1.33 Mangifera indica* L.	↓ Serum and BALF OVA-specific IgE, IL-4, and IL-5		[106]

*No.3.3.19 Morin*	↓ Inflammatory cells ↓ MMP-9 expression ↓ BALF total IgE, TNF-*α*, IL-4, IL-5, IL-13 ↓ Lung eotaxin1, MCP-1, IL-8, and ICAM-1 expression regulation of MAPK signaling pathway ↓Lung IgE, COX-2, IL-4, IL-6, and LTB-4 expression modulation of SUMF2/IL-13 and BLT2/NF-*κ*B signaling pathways	↓ Lung MDA ↓ Lung ROS ↓ NO ↓ Histamine release	[107–109]

*No.3.1.36 Moringa oleifera Lam*		↓ Serum histamine concentration	[110]

*No.3.3.20 Myrtenol*	↓ BALF, lung, and serum TNF-*α*, IL-1*β* ↑ BALF, lung, and serum IL-10, IFN-*γ*	↓ BALF, lung, and serum MDA ↑ BALF, lung, and serum SOD, GPX	[111, 112]

*No.3.3.22 Naringenine*	↓ Serum IgE ↓ BALF CCL11, CCL5, IL-4, IL-13 inhibition NF-*κ*B activity		[47, 113, 114]

*No.3.1.37 Nigella sativa*	↓ BALF IL-2, IL-6, IL-4, IL-5, IL-13 ↑ BALF IFN-*γ* ↓ Serum IgE, OVA-specific IgE, IgG ↓ BALF eosinophils ↓ PGE2	↓ Histamine release	[115–117]

*No.3.1.39 Ocimum basilicum*	↓ BALF IL-4, IgE, TP, PLA2 ↑ BALF IFN-*γ* ↓ Serum WBC	↓ Serum NO_2_, NO_3_, MDA ↑ Serum SOD, CAT, thiol	[118, 119]

*No.3.1.41 Phyllanthus amarus*	↓ TNF-*α*, IL-1*β*, IL-4, IL-6, TGF-*β*, IgE, Nrf2	↓ iNOS	[120, 121]

*No.3.1.43 Pimpinella anisum* L.	↓ IL-1*β*, IL-8	↓ NO-cGMP pathway	[50, 122]

*No.3.1.44 Pistacia alantica*	↓ IgE, IL-4, IL-5, IL-17 ↑ IFN-*γ*, IL-10, and TGF-*β*		[123]

*No.3.1.45 Portulaca oleracea*	↓ BALF IgE, PLA2, TP		[124]

*No.3.3.25 Quercetin*	↓ BALF eosinophil count, IL-4, IL-5, eotaxin ↑ BALF IFN-*γ* ↓ Lung and BALF IL-25, IL-33 ↓ Lung P-selectin, GATA3, MMP-9 expression ↓ NF-*κ*B activation ↑ Lung T-bet expression	↓ BALF EPO activity	[125–128]

*No.3.3.24 Qingfei Yihuo Wan*	↓ Serum IL-4, IL-5, IL-13, IL-1*β* ↓ Lung NF-*κ*B and PKC expression modulation of mTOR signaling pathway		[129, 130]

*No.3.3.26 Resveratrol*	↓ BALF IL-4, IL-5, IL-6, IL-17, TNF-*α*, TGF-*β* ↓ Inflammatory cells ↓ Serum IgE, IgG modulation of INPP4A inhibition of PI3K-Akt signaling pathway ↓ Lung TGF-*β* expression ↑ Lung FOXP3 ↑ Lung PTEN, SIRT1 expression inhibition of HMGB1/TLR4/NF-*κ*B pathway	↑ Keap 1/Nrf2 antioxidant defense system	[131–134]

*No.3.3.27 Rosmarinus officinalis*	↓ BALF IL-4, PLA2, TP, IgE ↑ BALF IFN-*γ*		[135]

*No.3.1.47 Sambucus nigra*	↓ BALF IL-4, IL-5, IL-13, IgE	↓ Lung MDA ↑ Lung SOD, CAT	[136]

*No.3.1.48 Sesame*	↓ Lung IL-6, IL-1*β* ↓ Serum IgE	↓ Lung NO_2_	[137]

*No3.1.49 Sophora flavescens*	↓ inflammatory cells ↓ IL-4, IL-5, IL-6, IL-13, TNF-*α*, eotaxin		[138]

*No.3.1.51 Thyme*	↓ IL-4, IL-5, IL-13, OVA-specific IgE, NF-*κ*B activation	↓ NO, hydrogen peroxide, MDA, isoprostane, and carbonyl group ↑ SOD, Gpx	[139, 140]

*No.3.1.53 Urtica dioica*	↓ BALF inflammatory cells, IL-4	↓ BALF MDA ↑ Serum and BALF SOD, GPX, GSH	[141]

*No.3.3.31. Vanillic acid*	↓ IL-4, IL-5, IL-13, TNF-*α*	↓ MDA, ROS ↑ SOD, GPX, GSH	[142]

*No.3.3.32. Vitexin*	↓ BALF IL-4, IL-5, IL-13 ↓ Serum IgE		[143]

*No.3.1.57 Vitis vinifera* L	↓ BALF and serum inflammatory cells, IL-4, IL-5, IL-1*β*, TNF-*α*, LTD-4, IgE	↓ BALF and serum NO, NO_2_, histamine	[144]

*No.3.1.58 Zingiber officinale*	↓ NF-*κ*B signaling, IL-2, IL-4, TNF-*α* ↑ PDE inhibition ↓ Lung IL-4, IL-5 expression ↓Serum IgE		[145, 146]

The number in the first column refers to the paragraph in the text describing the plant/compound characteristics.

## Abbreviations

AHR: Airway hyperresponsiveness
BALF: Bronchoalveolar lavage fluid
CAT: Catalase
CCR: C-C chemokine receptor
COX: Cyclooxygenase
EF: Expiratory flow
FEF: Forced expiratory flow
EPO: Erythropoietin
ET: Endothelin
FEV1: Forced expiratory volume in the first second
FOXP3: Forkhead box P3
GATA3: GATA binding protein 3
FVC: Forced vital capacity
GPx: Glutathione peroxidase
GSH: Glutathione
HDAC2: Histone deacetylase 2
ICAM-1: Intercellular adhesion molecule 1
IFN-*γ*: Interferon-gamma
IgE: Immunoglobulin E
IgG: Immunoglobulin G
iNOS: Inducible nitric oxide synthase
JNK: Janus kinase
5-LO: 5-lipoxygenase
LTC4: Leukotriene C4
MDA: Malondialdehyde
MAPK: Mitogen-activated protein kinase
MEF: Maximum expiratory flow
MMP-9: Matrix metalloproteinase 9
MPO: Myeloperoxidase
MVV: Maximal voluntary ventilation
NALF: Nasal lavage fluid
NF-*κ*B: Nuclear factor kappa B
NO: Nitric oxide
PAF: Platelet activator factor
PDE: Phosphodiesterase
PEF: Peak expiratory flow
PIF: Peak inspiratory flow
PEFR: Peak expiratory flow rate
PFT: Pulmonary function test
PLA2: Phospholipase A2
ROS: Reactive oxygen species
SOD: Superoxide dismutase
TNF: Tumor necrosis factor
TLR4: Toll-like receptor 4
TV: Tidal volume
MMEF: Maximal midexpiratory flow
Nrf2: Nuclear factor erythroid 2-related factor 2
TGF-*β*: Transforming growth factor-*β*
TIMP-1: Tissue inhibitor of metalloproteinase 1
TP: Total protein
VCAM-1: Vascular adhesion molecule 1.

## Supplementary Materials

Supplementary Materials The studies related to the plants with potentially antiasthmatic effects are described in the supplementary file. Figure 1 supplement: pictures of the plants with potential anti-asthmatic effects. In the text where plant features are described, the names of plants are arranged alphabetically. Table 1 supplement: plants or herbal compounds with potential anti-asthmatic effects in animal/organ/tissue studies, and the related mechanisms. The number in the first column refers to the paragraph in the text describing the plant characteristics.
