# Supplementary material for: An Updated Comprehensive Review of Plants and Herbal Compounds with Antiasthmatic Effect
Source: Evid Based Complement Alternat Med. 2024 Feb 8;2024:5373117. doi: 10.1155/2024/5373117 (PMC11390241; doi:10.1155/2024/5373117)
Supplement: Supplementary Materials — The studies related to the plants with potentially antiasthmatic effects are described in the supplementary file. Figure 1 supplement: pictures of the plants with potential anti-asthmatic effects. In the text where plant features are described, the names of plants are arranged alphabetically. Table 1 supplement: plants or herbal compounds with potential anti-asthmatic effects in animal/organ/tissue studies, and the related mechanisms. The number in the first column refers to the paragraph in the text describing the plant characteristics. [file 5373117.f1.docx]

**Supplement file:**

**An updated comprehensive review of plants and herbal compounds with anti-asthmatic effect**

**3.2. Plants with potentially anti-asthmatic effects**

Although how some herbs affect asthma remains to be directly studied, but due to their anti-inflammatory, anti-oxidative and bronchodilator effects, they may potentially have anti-asthmatic effects, subject to these effects to be investigated in future studies. The pictures of these plants are presented in Figure 1 supplement, and the studies related to these plants are described below. Table 1 supplement summarizes the properties of these plants pertaining to their anti-inflammatory, anti-histaminic, and bronco-dilator mechanisms.

**Figure 1** **supplement** **here**

**3.2.1. *Aerva lanata* Linn. *(Amaranthaceae)***

A prostrate or erect weed that commonly grows on the wayside, *Aerva lanta (A. lanta)*, is commonly found in parts of India with warmer climate. In the isolated tracheal chain preparation model in goats, the bronchodilator effects of the alcoholic extract of the plant have been demonstrated at 100 μg/mL [19]. It also at 30 and 60 mg/kg oral doses caused mast cell degranulation in mice in comparison to chlorpheniramine maleate (10 mg/kg) [1, 2].

**3.2.2. *Ageratum conyzoides* Linn.**

This annual herbaceous member of the *Asteraceae* family (Compositae), is native to the subtropical and tropical areas of the world, including tropical America [2]. The anti-inflammatory properties of this plant have been identified in animal models [2]. Achola *et al*. showed the broncho-dilating effect of *Ageratum conyzoides* extract on the isolated guinea-pig trachea [3].

**3.2.3. *Ajuga chamaepitys* Linn.**

This flowering member of the *Lamiaceae* family [4] has demonstrated strong anti-inflammatory and anti-oxidant activity of this plant [5-7]. Its inflammation-reducing action was exerted via cyclooxygenase (COX) inhibition [7].

**3.2.4. *Aloe vera* Linn.**

This species with succulent leaves is a perennial evergreen plant originally from the Arabian Peninsula, able to survive in arid, semi-tropical, tropical climates worldwide [8]. The anti-inflammatory and antioxidant effects of this plant makes it suitable to treat symptoms of asthma [5].

**3.2.5. *Anacyclus pyrethrum* Linn Lag. (Chamomile)**

Known by the by Moroccan people as “Tigandizt” or “Aqar-qarha”, this North African native’s roots are commonly employed to treat paralysis, neuralgia, respiratory problems, colds, sciatica, and rheumatism [5, 9]. Its aqueous and methanolic extracts have antioxidant, antinociceptive, and anti-inflammatory effects [5].

**3.2.6. *Andrographis paniculata (Acanthaceae)***

The inhibitory effect of the main active component of this plant, andrographolide, on nuclear factor (NF) kappa B activity has been demonstrated [10]. Asthma development has been associated with recurring NF-Kappa B activation.

**3.2.7. *Asystasia gangetica (Acanthaceae)***

Many Nigerians manage their respiratory problems using the therapeutic qualities of *A. gangetica* [2]. Akah *et al*. tested the methanolic, ethyl acetate, and hexane extracts of *A. gangetica* leaves on albumin-induced acute inflammation in guinea pig tracheae for possible anti-asthmatic activity [11]. Moreover, broncho-spasmolytic activity of the extract and fractions of *A. gangetica* leaves have been shown by Ezike and co-investigators [12].

**3.2.8. *Bacopa monnieri (Scrophulariaceae)***

This perennial creeping plant is a native of Australian, South and North American, Asian, African, European, and east and south Indian wetlands. The results of Samiulla, *et al*.’s evaluation of *B. monnieri* leaf water, methanol petroleum ether, and chloroform extracts showed significant mast cell degranulation inhibition in all the extracts at 10 mg/ml concentration [13].

**3.2.9. *Brassica nigra* (black mustard)**

The dark brown or black seeds this annual plant is cultivated for are known as black mustard and used as a spice. Due to the anti-inflammatory and antioxidant effects reported for this plant, it can be an option for the studies for the treatment of asthma [5, 14].

**3.2.10. *Cassia sophera (caesalpiniaceae)***

*C. sophera* is considered a traditional treatment for bronchitis, and at concentrations of 250, 500 and 750 mg/kg, the ethanol, ethyl acetate, and chloroform fractions isolated from leaves have been shown to possess significant protective qualities against bronchoconstriction in guinea pigs with histamine-induced bronchospasm and reduced eosinophilia [15].

**3.2.11. *Casuarina equisetifolia* Linn*. (Casuarinaceae)***

*Casuarina equisetifolia*, the Australian pine tree or whistling pine tree, is a she-oak species of the genus *Casuarina*. Histamine-induced contraction of the trachea is inhibited by the antihistaminic activity of its bark and wood methanol extract at the concentration of 10–80 mcg/ml, and it has also been found to reduce degranulation in mast cells at 100 mg/kg [16].

**3.2.12. *Cheilocostus speciosus* (J. Koenig)**

In traditional medicine, respiratory problems are treated with the rhizome of this plant, and the inflammation-reducing activity of the rhizome’s ethanol extract has been reported in rats [17]. eNOS expression is increased and histological changes, iNOS expression, inflammation, and oxidative stress are decreased by the steroid sapogenin, diosgenin, found in copious amounts in the rhizome, in pulmonary hypertension induced by monocrotaline in rats [18]. In addition, TNF-α generation induced by LPS in RAW 264.7 macrophages is inhibited by diosgenin with efficacy comparable to methotrexate [19].

**3.2.13. *Cinnamomum citriodorum* (thwaites)**

This plant belongs to a species of evergreen aromatic plant *Lauraceae.* The active ingredient in the essential oil of this plant, citronellol, is known to inhibit the production of PGE2 and NO by inhibiting iNOS activity [20, 21]. Citronellol activates peroxisome proliferator-activated receptors α and γ that are expressed in the airways, leading to dilatation of the bronchi [22, 23].

**3.2.14. *Cissus quadrangularis* Linn.**

*Cissus quadrangularis* is a plant native to India's warm regions, whose roots powder is used for healing of the bone fractures. Studies have shown that its methanolic extract has anti-inflammatory effects [24] due to the presence of flavonoids, especially luteolin and β-sitosterol [25]. The β-sitosterol can reduce MPO enzymes in the inflamed tissues. Anti-inflammatory activity against COX-2 have also been reported from methanolic and dichloromethane extracts of the plant stems [24]. It suppresses NF-κB activation and induces the heme-oxygenase-1 (HO-1) gene, inhibiting iNOS expression and NO production in macrophages. The smooth muscles of the airway have been found to relax *in vitro* by the inhibition of this enzyme [26, 27].

**3.2.15*. Citrullus colocynthis***

The activation of nicotinamide adenine dinucleotide phosphate (NADPH) oxidase is inhibited as a result of the peeling of inhibited ROS production in the neutrophils by *Citrullus colocynthis* aqueous extract *in vitro*. Lung inflammation induced by LPS was also reduced in mice, leading to a reduction in neutrophil content, total cell count, and protein concentration in the BALF [28]. In addition, the anti-inflammatory activity of *C. colocynthis* pulp hydro-alcoholic extract, has been demonstrated in obese mice. This is probably achieved by down-regulating the pro-inflammatory cytokines IL-6 and TNF-α [29].

**3.2.16. *Cnidium monnieri* (*Umbelliferae*)**

In Chinese traditional medicine, suppurative dermatitis, impotence, and pain in female genitalia, are treated relying on the anti-pruritogenic qualities of *Cnidium monnieri*. Its ethanol extract has been reported to have anti-allergic activity in Matsuda *et al*.’s study of passive cutaneous anaphylaxis in rats [30].

**3.2.17. *Commiphora myrrha* (Nees)**

This plant, identified as Balsamdendron myrrha and Commiphora, is a short tree or large shrub with many irregulars [31, 32]. In the past, Iranian, Chinese, Greek, and Arab physicians have used oleo-gum-resin of this plant to treat lung problems such as respiratory infections, chronic cough, diphtheria, rhinitis, tonsillitis, colds, and bronchitis [33]. PGE2, NO, TNF-α, and IL-1β production is inhibited by the ethanolic extract of *C. myrrha* gum through the regulation of COX-2 and iNOS gene expression in macrophages. Another gum resin constituent, dehydroabietic acid, decreases the of pro-inflammatory cytokine and chemokines production, by playing the role of a PPARα and γ agonist , including NO, TNF-α, and MCP-1 by macrophages. Shin and fellow researchers showed that myrrha reduces histamine release from stimulated human mast cells [33].

**3.2.18. *Crataegus mexicana* (Hawthorn)**

This is a hawthorn species native to parts of Guatemala and the mountains of Mexico . Arrieta *et al.* showed the relaxant effect of *Crataegus mexicana* leaves on the isolated tracheal rings of guinea pig [34].

**3.2.19. *Cuminum cyminum* (Cumin)**

A flowering member of the *Apiaceae* family, *C. cyminum* is native to the Middle East territory. Boskabady *et al*. demonstrated the antitussive role of *Cuminum cyminum* and its relaxant effect on the guinea pig tracheal chains [35, 36].

**3.2.20. *Datura stramonium***

This is a flowering species from the nightshade family *Solanaceae*. Pretorius *et al*. declared the potential role of *Datura stramonium* in asthma treatment due to its anticholinergic effects [37].

**3.2.21. *Ecballium elaterium* Linn. (Squirting cucumber)**

The *Cucurbitaceae* family contains a single species, *Ecballium elaterium* which is a genus of flowering plants [38]. The anti-inflammatory qualities of *E. elaterium* have been reported in studies. The symptoms in a rabbit model of rhinosinusitis were significantly reduced by the aqueous extract of *E. elaterium* administered topically, as it is able to diminish NO metabolite concentration and NOS enzyme activity [5, 39]. A pilot study clinically confirmed these findings in patients with rhinosinusitis [40].

**3.2.22. *Elephantopus scaber* Linn.**

*Elephantopus scaber* is a tropical species of flowering plant in the sunflower family. Anti-asthmatic potential of the E. scaber may be due to its antihistaminic, anticholinergic and mast-cell-stabilizing properties [41].

**3.2.23. *Foeniculum vulgare* (Fennel)**

*Foeniculum vulgare* is a flowering plant species in the carrot family. Boskabady *et al*. showed that this plant has relaxant effects on isolated guinea pig tracheal chains [42], implying its potential anti-asthmatic effect.

**3.2.24. *Hemidesmus indicus (Asclepiadaceae)***

Bhujbal *et al*. revealed that at concentrations of 25, 50 and 100 mg/kg, the alcoholic extract of the roots of this common Indian twining shrub possesses a relaxant effect in isolated goat tracheal chain preparation [43].

**3.2.25. *Lippia alba***

Carvalho *et al*. demonstrated that the essential oil of this flowering member of the *Verbenaceae* family and its major compound citral have antispasmodic effects on the smooth muscles of the trachea isolated from Wistar rats. It is believed that L-type voltage-operated calcium channels are blockaded by this agent to achieve this effect [44].

**3.2.26. *Melilotus officinalis* Linn. (Clover)**

*Melilotus officinalis*, known as yellow sweet clover, is a species of legume with a variety of anti-inflammatory and antioxidant effects [45, 46], which makes it a therapeutic option for asthma treatment in future studies.

**3.2.27. *Operculina turpethum* Linn. (Turpeth)**

*Operculina turpethum,* a plant in the morning glory family, is known commonly as turpeth. It has been demonstrated that *Operculina turpethum* has bronchodilator [47] and antioxidant activity [48, 49]. Accordingly, the plant seems to have the potential to produce anti-asthmatic effects.

**3.2.28. *Peganum harmala* Linn.**

*Peganum harmala* is the only salt-tolerant perennial herb in the *Peganum* genus of the family *Zygophyllaceae* [50]. It has been shown that ethanolic extract of *Peganum harmala* seeds offers mast cell protection against degranulation [51]. The inflammation- and oxidation-reducing effects of this plant have also been proven [52]. Liu *et al*. reported bronchodilatory, antitussive, and expectorant effects for the flavonoid fraction of this plant and the quinazoline alkaloids isolated from its aerial parts in guinea pigs and mice [5, 53, 54].

**3.2.29. *Piper betel* Linn.**

This is a traditional cure for rheumatism, asthma, pruritus, cold and cough. At concentrations of 100 and 200 mg/kg, the aqueous and ethanolic extracts of the leaves act as a bronchodilator on tracheal chain contraction and bronchoconstriction induced by histamine in guinea pigs [55].

**3.2.30. *Ruta graveolens* Linn.**

Known as rue, this is a Ruta species native to the Balkan peninsula. Its hydroalcoholic extract has exhibited relaxant effects on rat tracheal smooth muscles. Ionic current blockade through a noncompetitive antagonistic mechanism is probably involved [56]. Studies on the contraction induced in the isolated guinea pig trachea by histamine have confirmed the significant relaxant effects (IC50 = 0.42 mM) of the quinolone alkaloids abundantly found in the plant [5, 57, 58].

**3.2.31. *Striga orobanchioides* benth. *(Scrophulariaceae)***

The mast cell stabilizing and antihistaminic activities of the ethanolic and aqueous extracts of the complete plant have been confirmed by disclosing their inhibition of contractions induced by histamine in the Guinea pig ileum at the 2.5–25 mcg/ml concentrations. It also inhibited degranulation of mast cells at concentrations of 100 and 200 mg/kg [59].

**3.2.32. *Woodfordia fruticosa***

*Woodfordia fruticosa* Kurz. (*Lythraceae*) is an ethno pharmacologically popular plant in the Indian medicinal system. Ghante *et al*. revealed the inflammation-reducing capability of the flower extracts of *Woodfordia fruticosa* [60].

**Table 1 supplement here**

**References**

[1]. D. Kumar, D. Prasad, J. Parkash, S. Bhatnagar and D. Kumar, "Antiasthmatic activity of ethanolic extract of Aerva lanata Linn," *Pharmacologyonline*, vol. 2, pp. 1075-1081, 2009.

[2]. D. J. Taur and R. Y. Patil, "Some medicinal plants with antiasthmatic potential: a current status," *Asian Pacific journal of Tropical Biomedicine*, vol. 1, no. 5, pp. 413-418, 2011.

[3]. K. Achola and R. Munenge, "Bronchodilating and uterine activities of Ageratum conyzoides extract," *Pharmaceutical Biology*, vol. 36, no. 2, pp. 93-96, 1998.

[4]. B. List, "Botanical Society of Britain and Ireland. Archived from the original (xls) on 2015-01-25," Ed., Retrieved 2014-10-17, 2007.

[5]. B. Javadi, A. Sahebkar and S. Ahmad Emami, "Medicinal plants for the treatment of asthma: A traditional Persian medicine perspective," *Current Pharmaceutical Design*, vol. 23, no. 11, pp. 1623-1632, 2017.

[6]. S. Turkoglu, I. Turkoglu, M. Kahyaoglu and S. Celık, "Determination of antimicrobial and antioxidant activities of Turkish endemic Ajuga chamaepitys (L.) Schreber subsp. euphratica PH Davis (Lamiaceae)," *Journal of Medicinal Plants Research*, vol. 4, no. 13, pp. 1260-1268, 2010.

[7]. R. Gautam, S. M. Jachak and A. Saklani, "Anti-inflammatory effect of Ajuga bracteosa Wall Ex Benth. mediated through cyclooxygenase (COX) inhibition," *Journal of Ethnopharmacology*, vol. 133, no. 2, pp. 928-930, 2011.

[8]. M. Gong, F. Wang and Y. Chen, "Study on application of arbuscular-mycorrhizas in growing seedings of Aloe vera,"*Journal of Chinese medicinal materials*, vol. 25, no. 1, pp. 1-3, 2002.

[9]. H. Manouze, O. Bouchatta, A. C. Gadhi, M. Bennis, Z. Sokar and S. Ba-M’hamed, "Anti-inflammatory, antinociceptive, and antioxidant activities of methanol and aqueous extracts of Anacyclus pyrethrum roots," *Frontiers in pharmacology*, vol. 8, pp. 598, 2017.

[10]. Z. Bao, S. Guan, C. Cheng, S. Wu, S. H. Wong, D. M. Kemeny, B. P. Leung and W. F. Wong, "A novel antiinflammatory role for andrographolide in asthma via inhibition of the nuclear factor-κB pathway," *American journal of respiratory and critical care medicine*, vol. 179, no. 8, pp. 657-665, 2009.

[11]. P. Akah, A. Ezike, S. Nwafor, C. Okoli and N. Enwerem, "Evaluation of the anti-asthmatic property of Asystasia gangetica leaf extracts," *Journal of Ethnopharmacology*, vol. 89, no. 1, pp. 25-36, 2003.

[12]. Ezike A, Akah P, Okoli C: Bronchospasmolytic activity of the extract and fractions of Asystasia gangetica leaves. International Journal of applied research in natural products 2008.

[13]. D. Samiulla, D. Prashanth and A. Amit, "Mast cell stabilising activity of Bacopa monnieri," *Fitoterapia*, vol. 72, no. 3, pp. 284-285, 2001.

[14]. A. E. Al Snafi, "The pharmacological importance of Brassica nigra and Brassica rapa grown in Iraq," *Jornal of Pharmaceutical Biology*, 2015.

[15]. D. Nagore, V. Ghosh and M. Patil, "Evaluation of antiasthmatic activity of Cassia sophera Linn," *Pharmacognosy magazine*, vol. 5, no. 19, pp. 109, 2009.

[16]. A. Aher, S. Pal, U. Patil, S. Yadav and S. Bhattacharya, "Evaluation of anthistaminic activity of Casuarina equisetifolia frost (Casuarinaceae)," *Pharmacologyonline*, vol. 1, pp. 1144-1149, 2009.

[17]. K. Binny, S. G. Kumar and T. Dennis, "Anti-inflammatory and antipyretic properties of the rhizome of Costus speciosus (koen.) sm," *Journal of Basic and Clinical Pharmacy*, vol. 1, no. 3, pp. 177, 2010.

[18]. L. A. Ahmed, Z. O. Al Arqam, H. F. Zaki and A. M. Agha, "Role of oxidative stress, inflammation, nitric oxide and transforming growth factor-beta in the protective effect of diosgenin in monocrotaline-induced pulmonary hypertension in rats," *European Journal of Pharmacology*, vol. 740, pp. 379-387, 2014.

[19]. S. Selim and S. Al Jaouni, "Anti-inflammatory, antioxidant and antiangiogenic activities of diosgenin isolated from traditional medicinal plant, Costus speciosus (Koen ex. Retz.) Sm," *Natural product research*, vol. 30, no. 16, pp. 1830-1833, 2016.

[20]. N. Leela, T. Vipin, V. Priyanka, K. Shafeekh and J. Rema, "Cinnamomum citriodorum Thw.–new source of citronellol," *Journal of Essential Oil Research*, vol. 24, no. 6, pp. 507-511, 2012.

[21]. Y.-W. Su, S.-H. Chao, M.-H. Lee, T.-Y. Ou and Y.-C. Tsai, "Inhibitory effects of citronellol and geraniol on nitric oxide and prostaglandin E2 production in macrophages," *Planta Medica*, vol. 76, no. 15, pp. 1666-1671, 2010.

[22]. L. N. Andrade and D. P. De Sousa, "A review on anti-inflammatory activity of monoterpenes," *Molecules*, vol. 18, no. 1, pp. 1227-1254, 2013.

[23]. A. Trifilieff, A. Bench, M. Hanley, D. Bayley, E. Campbell and P. Whittaker, "PPAR‐α and‐γ but not‐δ agonists inhibit airway inflammation in a murine model of asthma: in vitro evidence for an NF‐κB‐independent effect," *British journal of pharmacology*, vol. 139, no. 1, pp. 163-171, 2003.

[24]. A. Siddiqua and S. Mittapally, "A review on Cissus quadrangularis," *The Pharma Innovation*, vol. 6, no. 7, Part E, pp. 329, 2017.

[25]. A. Panthong, W. Supraditaporn, D. Kanjanapothi, T. Taesotikul and V. Reutrakul, "Analgesic, anti-inflammatory and venotonic effects of Cissus quadrangularis Linn," *Journal of Ethnopharmacology*, vol. 110, no. 2, pp. 264-270, 2007.

[26]. K. Srisook, M. Palachot, N. Mongkol, E. Srisook and S. Sarapusit, "Anti-inflammatory effect of ethyl acetate extract from Cissus quadrangularis Linn may be involved with induction of heme oxygenase-1 and suppression of NF-κB activation," *Journal of Ethnopharmacology*, vol. 133, no. 3, pp. 1008-1014, 2011.

[27]. A. M. Bhujade, S. Talmale, N. Kumar, G. Gupta, P. Reddanna, S. K. Das and M. Patil, "Evaluation of Cissus quadrangularis extracts as an inhibitor of COX, 5-LOX, and proinflammatory mediators," *Journal of Ethnopharmacology*, vol. 141, no. 3, pp. 989-996, 2012.

[28]. F. Zioud, A. B. Mahmoud, T. Boussetta, J. El-Benna and R. Bachoual, "An aqueous Citrillus colocynthis peel extract inhibits neutrophil reactive oxygen species production and attenuates lung inflammation in mice," *Journal of Medicinal Plants Research*, vol. 9, no. 31, pp. 829-837, 2015.

[29]. N. Sanadgol, S. Najafi, L. V. Ghasemi, G. Motalleb and J. Estakhr, "A study of the inhibitory effects of Citrullus colocynthis (CCT) using hydro-alcoholic extract on the expression of cytokines: TNF-α and IL-6 in high fat diet-fed mice towards a cure for diabetes mellitus," *Journal of pharmacognosy and phytotherapy*, vol. 3, no. 6, pp. 81-88, 2011.

[30]. H. Matsuda, N. Tomohiro, Y. Ido and M. Kubo, "Anti-allergic effects of cnidii monnieri fructus (dried fruits of Cnidium monnieri) and its major component, osthol," *Biological and Pharmaceutical Bulletin*, vol. 25, no. 6, pp. 809-812, 2002.

[31]. T. Shen, G.-H. Li, X.-N. Wang and H.-X. Lou, "The genus Commiphora: a review of its traditional uses, phytochemistry and pharmacology," *Journal of Ethnopharmacology*, vol. 142, no. 2, pp. 319-330, 2012.

[32]. I. Shameem, "Phytochemical & therapeutic potentials of Murr Makki (Commiphora myrrha): A review," *Indian J Appl Res*, vol. 8, no. 9, pp. 102-104, 2018.

[33]. R. ABUKHADER and A. R. AL TAWAHA, "Amazing Benefits of Myrrh," *International Journal of Pharmaceutical Research*, vol. 13, no. 2, 2021.

[34]. J. Arrieta, D. Siles-Barrios, J. García-Sánchez, B. Reyes-Trejo and M. E. Sánchez-Mendoza, "Relaxant effect of the extracts of Crataegus mexicana on guinea pig tracheal smooth muscle," *Pharmacognosy Journal*, vol. 2, no. 17, pp. 40-46, 2010.

[35]. M. Boskabady, S. Kiani and H. Azizi, "Relaxant effect of Cuminum cyminum on guinea pig tracheal chains and its possible mechanism (s)," *Indian Journal of Pharmacology*, vol. 37, no. 2, pp. 111, 2005.

[36]. M. H. Boskabady, S. Kiani, H. Azizi and T. Khatami, "Antitussive effect of Cuminum cyminum Linn. in guinea pigs," NIScPR Online Periodicals Repository , 2006.

[37]. E. Pretorius and J. Marx, "Datura stramonium in asthma treatment and possible effects on prenatal development," *Environmental Toxicology and Pharmacology*, vol. 21, no. 3, pp. 331-337, 2006.

[38]. G. Kloutsos, D. G. Balatsouras, A. C. Kaberos, D. Kandiloros, E. Ferekidis and C. Economou, "Upper airway edema resulting from use of Ecballium elaterium," *The Laryngoscope*, vol. 111, no. 9, pp. 1652-1655, 2001.

[39]. C. Uslu, R. M. Karasen, F. Sahin, S. Taysi and F. Akcay, "Effect of aqueous extracts of Ecballium elaterium rich, in the rabbit model of rhinosinusitis," *International journal of pediatric otorhinolaryngology*, vol. 70, no. 3, pp. 515-518, 2006.

[40]. E. E. Mazokopakis, C. M. Karefilakis and I. K. Starakis, "The safety and efficacy of the fruit juice of Ecballium elaterium in the treatment of acute rhinosinusitis," *The Journal of Alternative and Complementary Medicine*, vol. 15, no. 12, pp. 1273-1274, 2009.

[41]. R. Sagar and H. Sahoo, "Evaluation of antiasthmatic activity of ethanolic extract of Elephantopus scaber L. leaves," *Indian journal of pharmacology*, vol. 44, no. 3, pp. 398, 2012.

[42]. M. Boskabady and A. Khatami, "Relaxant effect of Foeniculum vulgare on isolated guinea pig tracheal chains," *Pharmaceutical biology*, vol. 41, no. 3, pp. 211-215, 2003.

[43]. S. Bhujbal, D. Kumar, R. Deoda, T. Deore and M. Patil, "ANTIASTHMATIC ACTIVITY OF ROOTS OF Hemidesmus indicus R. Br," *Pharmacologyonline*, vol. 1, pp. 209-216, 2009.

[44]. P. M. Carvalho, C. A. Macêdo, T. F. Ribeiro, A. A. Silva, R. E. Da Silva, L. P. de Morais, M. R. Kerntopf, I. R. Menezes and R. Barbosa, "Effect of the Lippia alba (Mill.) NE Brown essential oil and its main constituents, citral and limonene, on the tracheal smooth muscle of rats," *Biotechnology reports*, vol. 17, pp. 31-34, 2018.

[45]. A. E. Al-Snafi, "Chemical constituents and pharmacological effects of Melilotus Officinalis-A review," *IOSR Journal of Pharmacy*, vol. 10, no. 1, pp. 26-36, 2020.

[46]. L. Pleşca‐Manea, A. E. Pârvu, M. Parvu, M. Taaˇmaş, R. Buia and M. Puia, "Effects of Melilotus officinalis on acute inflammation," *Phytotherapy research*, vol. 16, no. 4, pp. 316-319, 2002.

[47]. H. Shareef, G. H. Rizwani, S. R. Mandukhail, N. Watanabe and A. H. Gilani, "Studies on antidiarrhoeal, antispasmodic and bronchodilator activities of Operculina turpethum Linn," *BMC complementary and alternative medicine*, vol. 14, no. 1, pp. 1-7, 2014.

[48]. M. I. Ezeja, S. O. Onoja, Y. N. Omeh and C. A. Chibiko, "Analgesic and antioxidant activities of the methanolic extract of Operculina turpethum leaves in mice," *International Journal of Basic and Clinical Pharmacology*, vol. 4, pp. 453-457, 2015.

[49]. T. Ahmad, M. K. Husain, M. Tariq, J. I. Siddiqui, M. Khalid, M. W. Ahmed and M. H. Kazmi, "A review on Operculina turpethum: A potent herb of Unani system of medicine," *Journal of Pharmacognosy and Phytochemistry*, vol. 6, no. 1, pp. 23-26, 2017.

[50]. Y. Li, Q. He, S. Du, S. Guo, Z. Geng and Z. Deng, "Study of methanol extracts from different parts of Peganum harmala L. using 1H-NMR plant metabolomics," *Journal of Analytical Methods in Chemistry*, vol. 2018, 2018.

[51]. A. Pathan and G. Vadnere, "Peganum harmala seeds: evaluation of antiasthmatic effect by using clonidine induced mast cell degranulation." *NeuroPharmacology Journal* ,Vol 1 , 2016.

[52]. J. Asgarpanah and F. Ramezanloo, "Chemistry, pharmacology and medicinal properties of Peganum harmala L," *African Journal of pharmacy and pharmacology*, vol. 6, no. 22, pp. 1573-1580, 2012.

[53]. W. Liu, Y. Wang, D.-d. He, S.-p. Li, Y.-d. Zhu, B. Jiang, X.-m. Cheng, Z.-t. Wang and C.-h. Wang, "Antitussive, expectorant, and bronchodilating effects of quinazoline alkaloids (±)-vasicine, deoxyvasicine, and (±)-vasicinone from aerial parts of Peganum harmala L," *Phytomedicine*, vol. 22, no. 12, pp. 1088-1095, 2015.

[54]. W. Liu, X. Cheng, Y. Wang, S. Li, T. Zheng, Y. Gao, G. Wang, S. Qi, J. Wang and J. Ni, "In vivo evaluation of the antitussive, expectorant and bronchodilating effects of extract and fractions from aerial parts of Peganum harmala linn," *Journal of Ethnopharmacology*, vol. 162, pp. 79-86, 2015.

[55]. N. Jawale, A. Shewale, G. Nerkar and V. Patil, "Evalution of antihistaminic activity of leaves of Piper betel Linn," *Pharmacologyonline*, vol. 3, pp. 966-977, 2009.

[56]. L. Águila, J. Ruedlinger, K. Mansilla, J. Ordenes, R. Salvatici, R. R. de Campos and F. Romero, "Relaxant effects of a hydroalcoholic extract of Ruta graveolens on isolated rat tracheal rings," *Biological Research*, vol. 48, no. 1, pp. 1-6, 2015.

[57]. J. Y. Salib, S. A. El-Toumy, E. M. Hassan, N. H. Shafik, S. M. Abdel-Latif and I. Brouard, "New quinoline alkaloid from Ruta graveolens aerial parts and evaluation of the antifertility activity," *Natural product research*, vol. 28, no. 17, pp. 1335-1342, 2014.

[58]. C.-H. Lee and H.-S. Lee, "Relaxant effect of quinoline derivatives on histamine-induced contraction of the isolated guinea pig trachea," *Journal of the Korean Society for Applied Biological Chemistry*, vol. 54, no. 1, pp. 118-123, 2011.

[59]. M. S. Harish, M. Nagur and S. Badami, "Antihistaminic and mast cell stabilizing activity of Striga orobanchioides," *Journal of Ethnopharmacoogyl*, vol. 76, no. 2, pp. 197-200, 2001.

[60]. A. Najda, A. Bains, P. Chawla, A. Kumar, S. Balant, M. Walasek-Janusz, D. Wach and R. Kaushik, "Assessment of Anti-Inflammatory and Antimicrobial Potential of Ethanolic Extract of Woodfordia fruticosa Flowers: GC-MS Analysis," *Molecules*, vol. 26, no. 23, 2021.
